# A haloarchaeal VapBC toxin-antitoxin system: regulatory mechanism and role in establishing population heterogeneity to facilitate survival of subpopulations under stress conditions

**DOI:** 10.1128/aem.00747-26

**Published:** 2026-07-20

**Authors:** Dan He, Yuqi Lin, Yanyan Zhang, Yang Liu, Ping Li, Xin Su, Xiaoyi Qu, Yaru Liu, Xianyi Shao, Hongbo Wen, Hongyi Luo, Yi Wu, Fei Gan, Xiao-Feng Tang, Bing Tang

**Affiliations:** 1 Hubei Key Laboratory of Cell Homeostasis, Wuhan University, College of Life Sciences 98436, Wuhan, Hubei, China; 2 Cooperative Innovation Center of Industrial Fermentation (Ministry of Education & Hubei Province), Wuhan, China; 3 State Key Laboratory of Virology, College of Life Sciences, Wuhan University 98436, Wuhan, China; Kyoto University, Kyoto, Japan

**Keywords:** haloarchaea, toxin-antitoxin system, VapBC, regulation, stress resistance

## Abstract

Toxin-antitoxin (TA) systems are ubiquitously distributed in bacteria and archaea and have been implicated in various functions such as plasmid maintenance, phage defense, biofilm formation, stress response, and persistence, but their regulatory mechanisms and roles in haloarchaea are largely unknown. In this study, we found that four of the eight putative VapBC TA systems of *Natrinema gari* J7-2 are functional in strain J7-2 and *Haloferax volcanii*. Among them, the NgVapBC1 system is composed of the antitoxin NgVapB1 and the toxin NgVapC1, and the two proteins could form a NgVapBC1 complex. The NgVapBC1 system could be transcriptionally autoregulated, wherein a pseudo-palindromic sequence in the *vapBC1* operon promoter acts as a negative *cis*-regulatory element, and the NgVapBC1 complex is a stronger repressor than NgVapB1. The qRT-PCR and *in vivo* toxicity analyses showed that strain J7-2 maintains a high *vapB1*/*vapC1* mRNA ratio through truncation of the *vapBC1* operon transcript within the toxin-coding region for preventing the synthesis of excess toxin NgVapC1 to release toxicity abnormally. Mutational analyses showed that differential start codon usage by *vapB1* and *vapC1* and a −4 nt overlap of their stop and start codons contribute to maintaining a higher *vapB1*/*vapC1* mRNA ratio, and the −4 nt overlap-mediated translational coupling of *vapB1* and *vapC1* would enable both post-transcriptional and translational regulation of the NgVapBC1 system. The phenotypic comparison of strain J7-2 and its Δ*vapBC1* mutant revealed that the NgVapBC1 system contributes to population heterogeneity of decline-phase rather than exponential-phase strain J7-2 and facilitates the survival of subpopulations under stress conditions.

**IMPORTANCE:**

Although TA systems are regarded as versatile modulators of prokaryotic cell fate, their *bona fide* physiological roles remain elusive. Moreover, so far, there is no report on the regulatory mechanism and function of haloarchaeal VapBCs, which constitute the major group of TA systems in haloarchaea. Our results demonstrate that a VapBC system of the haloarchaeon *Natrinema gari* J7-2 is regulated at multiple levels and mediates the establishment of population heterogeneity, thereby facilitating the survival of the strain J7-2 population as a whole in diverse and changing environments. This study provides the first experimental evidence of the regulatory mechanism of the biological role of the VapBC system in conferring fitness advantages to haloarchaea.

## INTRODUCTION

Toxin-antitoxin (TA) systems are ubiquitous and abundant in prokaryotes and consist of a toxin that inhibits an essential cellular process and an antitoxin that neutralizes its cognate toxin. The genes encoding antitoxin and toxin are usually organized as a bicistronic operon and commonly located in mobile genetic elements (MGEs), including plasmids, prophages, integrative and conjugative elements, or transposons, or constitute small genomic islets by themselves in chromosomes, often in multiple copies (1). TA systems are currently classified into eight types (I–VIII) based on the nature and mode of action of the antitoxin, while type II systems, of which both toxin and antitoxin are proteins, are probably the most prevalent (1, 2). Type II TA systems are generally autoregulated at the transcriptional level through a mechanism of conditional cooperativity: at low toxin/antitoxin ratios, the TA complex cooperatively binds to the promoter region to repress TA operon transcription, whereas at high toxin/antitoxin ratios, the excess toxin destabilizes the TA-operator complex to induce transcription (3, 4). Type II TA systems could also be regulated at the post-transcriptional level by limited cleavage of toxin mRNA (5, 6). In terms of post-translational regulation of type II systems, a long-lasting paradigm suggests that stressful conditions would stimulate preferential degradation of the antitoxin in the TA complex and promote the liberation of the toxin; however, recent studies challenge this view and suggest that one likely mechanism for toxin activation is conformational changes in the TA complex due to the binding of a third protein (1, 2, 7).

The virulence-associated proteins B and C (VapBC) system represents the largest subgroup of the type II TA systems (2, 8). VapC toxins belong to the PilT N-terminal (PIN) domain family with endoribonuclease activity (9), and several of them have been shown to cleave unique tRNA, rRNA, and mRNA species (10–12). VapB antitoxins consist of an N-terminal DNA-binding domain that autoregulates the TA operon and a C-terminal region that obstructs the VapC active site in the VapBC complex to neutralize the toxin (9, 13, 14). Diverse functions have been assigned to TA systems including VapBCs, such as plasmid maintenance, integrative MGE stabilization, phage defense, biofilm formation, stress response, and persistence, although the roles of TA systems in stress response and persistence remain controversial (2, 7, 15, 16). The elucidation of the biological functions of TA systems is still an ongoing challenge.

For archaea, VapBCs have been intensively studied in thermoacidophilic archaea and implicated in stress responses. For instance, *Saccharolobus solfataricus* (formerly *Sulfolobus solfataricus*) possesses 22 VapBCs, and the inactivation of the *vapBC6* operon by targeted gene disruption leads to heat-shock sensitivity and a heat-dependent reduction in growth rate, suggesting that VapBC6 could regulate thermophilicity of the archaeon (11, 17). Based on the evidence that three of the 12 VapCs of *Metallosphaera prunae* could degrade rRNA *in vitro*, it has been proposed that this thermoacidophilic archaeon employs VapBCs for post-transcriptional regulation under uranium stress (18). In *Sulfolobus acidocaldarius*, which has 18 VapBCs, gene deletion analyses show that VapBC14 is involved in regulating biofilm formation (19), while VapBC4 not only participates in regulating biofilm formation but also plays an important role in heat stress adaptation (12). Given that thermoacidophilic archaea lack versions of bacterial heat shock proteins (HSPs), including Hsp100, Hsp90, and the DnaK/DnaJ/GrpE system (20), it has been suggested that other strategies, such as TA systems, are involved in handling thermal stress (16). In addition, VapCs from hyperthermophilic archaea, such as *Pyrobaculum aerophilum* (21, 22), *Archaeoglobus fulgidus* (23), and *Pyrococcus horikoshii* (24), represent the earliest reported PIN domain proteins that have been structurally characterized by X-ray crystallography, which have provided valuable information essential for understanding the structure-function relationship of VapBCs (9).

Much less is known about the role of TA systems in haloarchaea. The MazF toxins of the MazEF type II TA system from *Haloquadra walsbyi* (25) and *Methanohalobium evestigatum* (26) act as sequence-specific mRNA interferases and could exert toxicity when expressed in *Escherichia coli*, but their physiological roles in haloarchaea remain unclear. The CreTA type VIII TA system functions as an addiction module that prevents the loss of its cognate CRISPR-Cas system in *Haloarcula hispanica* (27). The temperate haloarchaeal virus SNJ2 encodes a functional HicAB type II TA system, of which the HicA toxin is toxic to the host cell in the absence of the HicB antitoxin, but this TA system does not participate in the SNJ2 life cycle transition (28). To date, there is no report on the function of the VapBC system in haloarchaea, although VapBCs constitute the major group of haloarchaeal TA systems (29).

Haloarchaea thrive in hypersaline environments (e.g., solar salterns, salt lakes, and salt deposits) and are usually exposed to environmental stresses associated with changes in salinity, temperature, radiation, nutrient availability, etc. Notably, both haloarchaea and thermoacidophilic archaea lack Hsp100 and Hsp90, but the former possess the DnaK/DnaJ/GrpE system that is absent in the latter (20). It is interesting to investigate whether exerting the function of the VapBC system could serve as an alternative strategy for handling stresses in haloarchaea, as proposed for thermoacidophilic archaea (16).

The haloarchaeon *Natrinema gari* J7-2 was isolated from a salt mine in China (30, 31). *Nnm. gari* strains have also been isolated from Thai traditional salty fish sauce and used for degrading histamine in high salt-fermented fishery products (32). We have previously characterized three extracellular subtilases (SptA (33, 34), SptC (35), and SptE (36)) and three HtrA proteases (NgHtrA, NgHtrB, and NgHtrC) (37) from strain J7-2 and found that these proteases are important for the growth, survival, and the stress response of the haloarchaeon. For instance, SptA not only contributes to the growth-phase transition of strain J7-2 but also facilitates the viable cells to scavenge the dead cell-derived proteins under nutrient-limiting conditions (38–40). As an extracytoplasmic protein control system, the HtrA proteases are essential for the survival of strain J7-2 in response to stresses such as heat, high salinity, and toxic substances (37). Additionally, thermal and osmotic stresses promote the expression of DnaK in *Natrinema* sp. J7, implying the role of this chaperone in the stress response of the haloarchaeon (41). By analyzing the complete genome sequence of strain J7-2 (30), eight putative *vapBC* loci were identified on the chromosome of this haloarchaeon. Recently, one of the predicted antitoxins, namely NJ7G_2729, was found to potentially participate in cell division (42). It is unclear whether and how the eight predicted VapBCs actually function as TA systems and also play a role in the survival and stress response of strain J7-2. In this study, four of the eight putative VapBC systems of *Nnm. gari* J7-2 are experimentally confirmed to be functional in strain J7-2 and *Hfx. volcanii*, and we focused on NgVapBC1 to investigate its regulatory mechanism and physiological role. NgVapBC1 was found to be regulated at multiple levels and contribute to the establishment of population heterogeneity, which confers fitness advantages to strain J7-2.

## RESULTS

### Bioinformatics analysis identified eight putative VapBCs in *Nnm. gari* J7-2

Eight VapBC-encoding loci (Table 1) were identified on the chromosome of strain J7-2 by the online tool TAfinder (43). According to the prediction by IslandViewer (44), the locus encoding NJ7G_2321/4368 resides on a ~9.3 kb MGE, while those encoding NJ7G_3854/3855 and NJ7G_3856/3857 reside on another ~36.9 kb MGE; they show high sequence identity (80%–95%) to homologous TA pair loci on plasmids of other haloarchaea (Table 1). The loci encoding NJ7G_0345/0346, NJ7G_0582/0581, and NJ7G_3796/3795 also share high sequence identity (>70%) with plasmid-borne TA pair loci of other haloarchaea (Table 1), and the former two have a lower GC content than the average of the chromosome (Fig. S1), implying that the three *vapBC* loci are also located on MGEs. It appears that at least five of the eight *vapBC* loci may be propagated via horizontal gene transfer.

**TABLE 1:** Putative VapBCs in strain J7-2 and their homologs in haloarchaeal MGEs

	Putative VapBCs in strain J7-2			Homologous TA pairs in haloarchaeal MGEs^*a*^		
	Antitoxin	Toxin	Feature of gene locus region	Identity^*b*^ (%)	MGE (GenBank no.)	Strain
1	NJ7G_0345	NJ7G_0346	Lower GC content than the average of the chromosome	79	Plasmid pNMAG02 (CP001934)	*Natrialba magadii* ATCC 43099
2	NJ7G_0582	NJ7G_0581	Lower GC content than the average of the chromosome	71	Plasmid pHFX3 (CP172598)	*Haloferax* sp. S1W
3	NJ7G_2321	NJ7G_4368	A MGE (~9.3 kb) with six transposase/integrase genes	95	Plasmid unnamed1 (CP101874)	*Natrinema thermotolerans* A29
4	NJ7G_2439	NJ7G_2438				
5	NJ7G_2729	NJ7G_2730	NJ7G_2729 potentially participates in cell division (42)			
6	NJ7G_3796	NJ7G_3795^*c*^		74	Plasmid pHALXA01 (CP002840)	*Halopiger xanaduensis* SH-6
7	NJ7G_3854^c^	NJ7G_3855	A MGE (~36.9 kb) with one transposase/integrase gene	80	Plasmid pHsi55 (CP073370)	*Haloarcula marismortui* ATCC 33800
8	NJ7G_3856	NJ7G_3857	Co-locates with NJ7G_3854/3855 in the same MGE	92	Plasmid pNPA200 (CP040638)	*Natrinema pallidum* BOL6-1

^*a*^ The information of haloarchaeal MGEs with TA pairs that show highest sequence identities to corresponding TA pairs of strain J7-2 is shown.

^*b*^ Nucleotide sequence identity between the TA pair-encoding region of strain J7-2 and its homolog in haloarchaeal MGE.

^*c*^ A 1-bp deletion in the gene causes an N-terminal truncation of NJ7G_3795, and a 1-bp insertion in the gene leads to an N-terminal truncation of NJ7G_3854.

**Fig 1:**
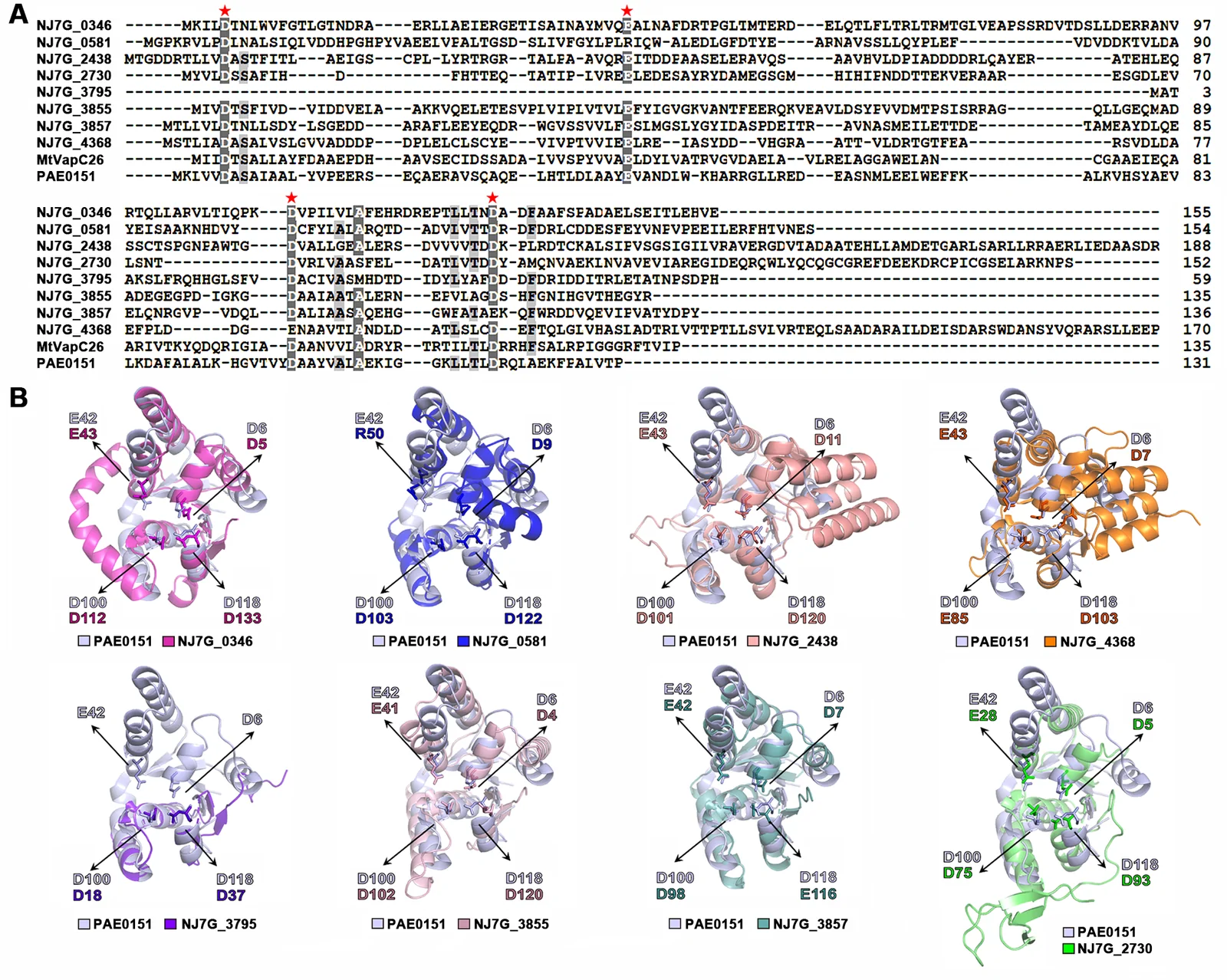
Primary and tertiary structures of putative VapCs of strain J7-2. (**A**) Amino acid sequence alignment of the eight VapCs of strain J7-2, MtVapC26 (WP_070895652, 5X3T), and PAE0151 (Q8ZZP3). The asterisks indicate four conserved acidic residues in the active site of VapC toxins. (**B**) Superimpositions of predicted structure models of VapCs of strain J7-2 with the crystal structure of PAE0151 (2FE1). The conserved acidic residues in the active site are indicated.

Among the eight putative VapCs, NJ7G_3795 is an N-terminal truncated form of VapC, while the other seven all possess three or four conserved acidic residues in the active site of VapC toxins (Fig. 1A). Although these putative VapCs show very low sequence identity (~10%–19%) to known homologs such as PAE0151 from *P. aerophilum* (22) and MtVapC26 from *Mycobacterium tuberculosis* (45) (Fig. 1A), the overall structures of all of them except NJ7G_3795, predicted by AlphaFold 3 (46), are well superimposed with the crystal structure of PAE0151 (22) (Fig. 1B).

**Fig 2:**
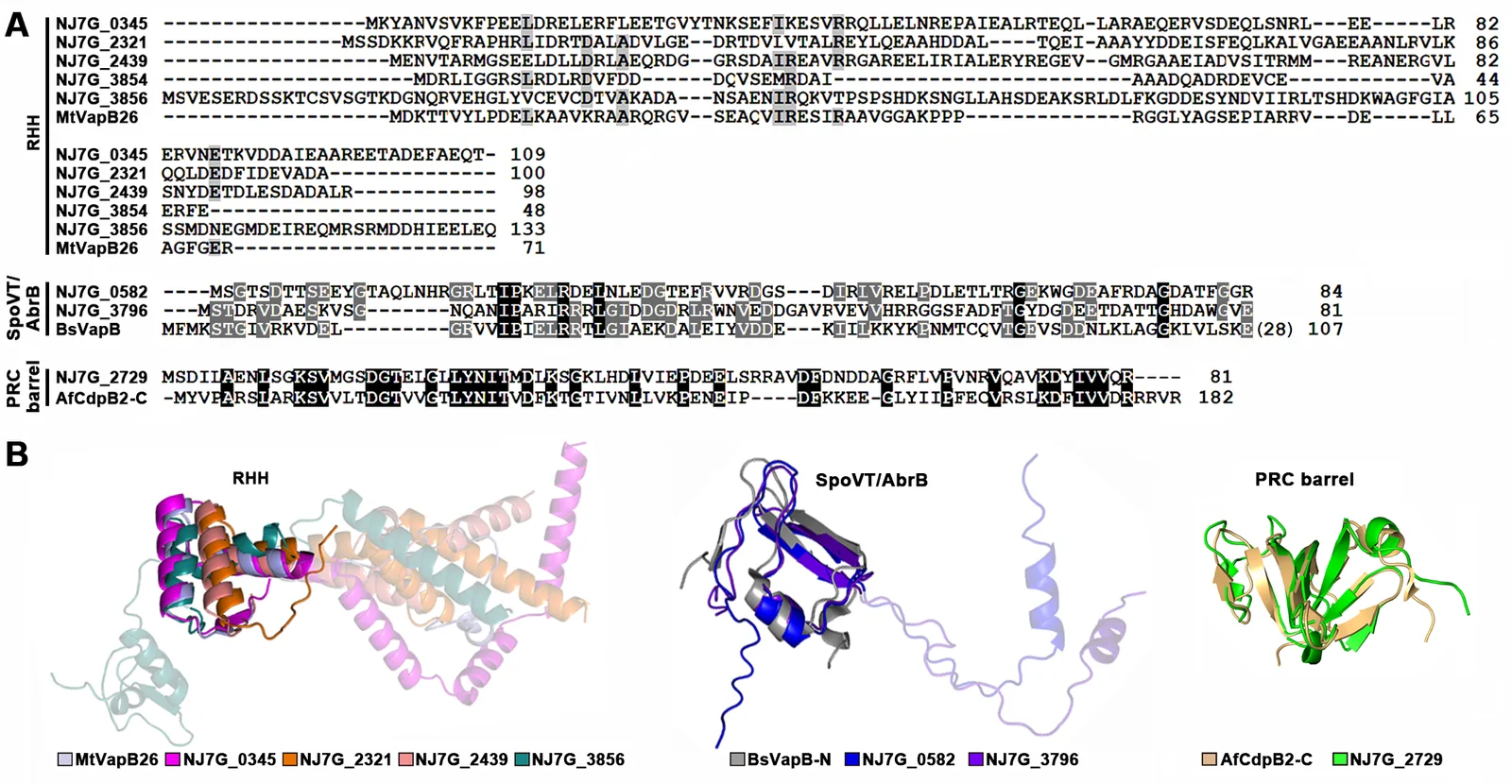
Primary and tertiary structures of putative VapBs of strain J7-2. (**A**) Amino acid sequence alignment of the VapBs of strain J7-2 with MtVapB26 (WP_003910158), BsVapB (WP_080332445), and AfCdpB2-C (WP_010877866), respectively. (**B**) Superimpositions of predicted structure models of VapBs of strain J7-2 with the structure models of MtVapB26 (5X3T), BsVapB (2K1N), and AfCdpB2-C (8QZO), respectively.

According to their similarities to known homologs in primary and tertiary structures, the putative VapBs of strain J7-2 can be classified into three groups, which contain a ribbon-helix-helix (RHH) domain, a SpoVT/AbrB domain, and a photosynthetic reaction center (PRC) barrel domain, respectively (Fig. 2). For the members of the first group, despite sharing only ~10%–17% sequence identity with MtVapB26 of *M. tuberculosis* (Fig. 2A), NJ7G_0345, NJ7G_2321, NJ7G_2439, and NJ7G_3856 all have an N-terminal domain that is well superimposed with the DNA-binding RHH domain of MtVapB26 (45) (Fig. 2B). NJ7G_3854 is an N-terminal truncation of RHH domain-containing VapB, with a size only about half those of its homologs (Fig. 2A). In the second group, NJ7G_0582 and NJ7G_3796 also show very low sequence identity (~15%–17%) to BsVapB of *Bacillus subtilis*, but their N-terminal domains are well superimposed with the DNA-binding SpoVT/AbrB domain of the latter (47) (Fig. 2B). For NJ7G_2729, it shares 41% sequence identity with the C-terminal YlmC domain (AfCdpB2-C) of the cell division protein B2 (CdpB2) of *A. fulgidus* (Fig. 2A) and adopts a PRC barrel structure highly similar to that of the latter (48) (Fig. 2B).

### Heterologous expression and gene deletion analyses revealed four functional VapBCs

**Fig 3:**
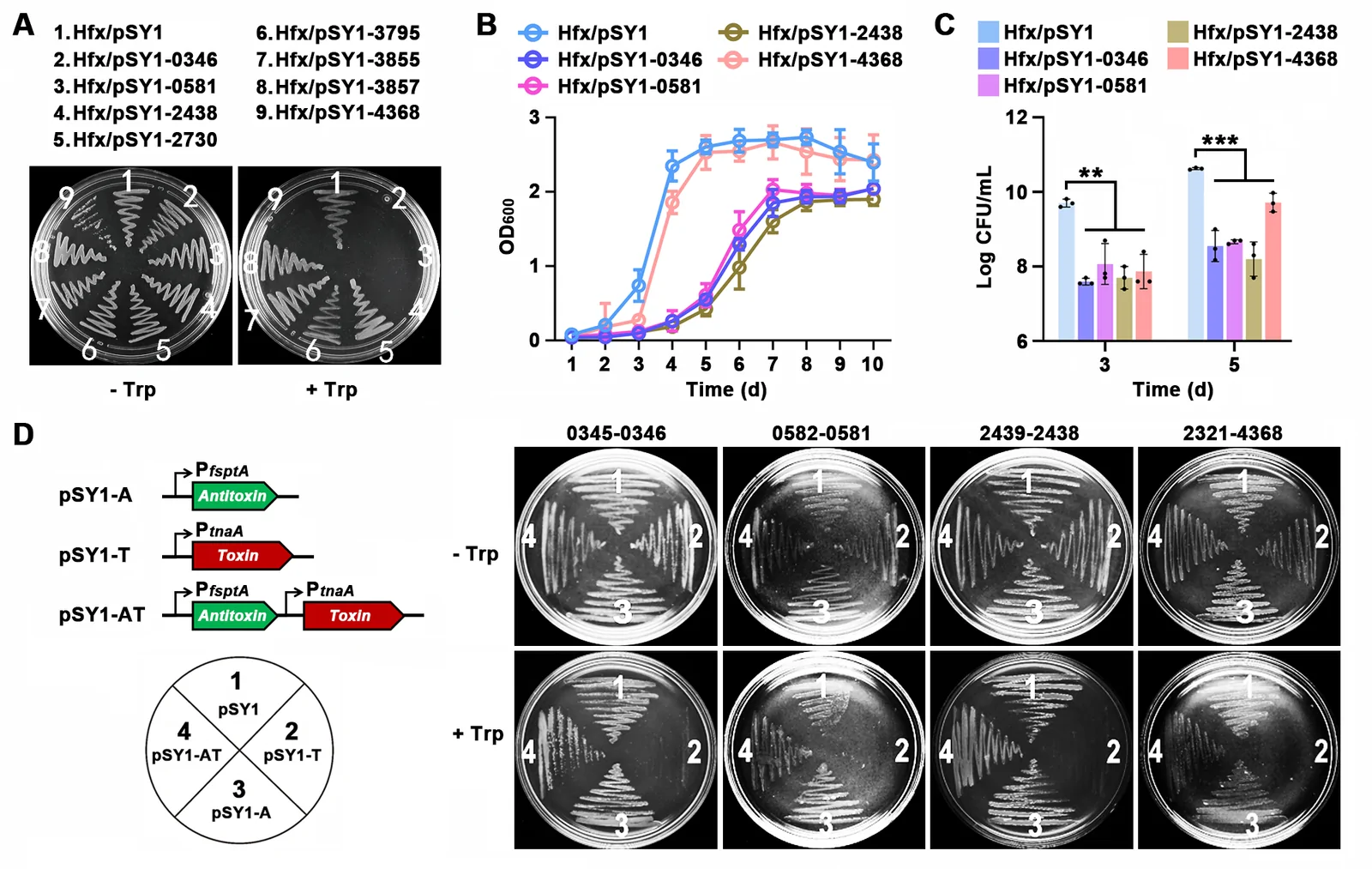
Functional analyses of VapBCs of strain J7-2 in *Hfx. volcanii*. (**A–C**) Effects of VapCs of strain J7-2 on the growth of *Hfx. volcanii. Hfx. volcanii* strain carrying the blank vector (pSY1) or the expression plasmid for target protein was streaked on Hv-Cab agar plate without (−) or with (+) 1 mM tryptophan (Trp) and grown at 37°C for 4 days (**A**). Target strains were cultivated at 37°C in 18% Hv-Cab liquid medium supplemented with 1 mM tryptophan and novobiocin (0.3 μg/mL). At the indicated time points, samples were taken and subjected to OD_600_ measurement (**B**) and viable cell counting on Hv-Cab agar plate at 37°C (**C**). The data are expressed as means ± SDs from biological triplicates (***, *P* < 0.001; **, *P* < 0.01). (**D**) Neutralization of VapCs by VapBs in *Hfx. volcanii. Hfx. volcanii* strain carrying the expression plasmid for antitoxin alone, toxin alone, or both antitoxin and toxin was streaked on novobiocin (0.3 μg/mL)-containing 18% Hv-Cab agar plates without (−) or with (+) 1 mM tryptophan and grown at 37°C for 4 days. Schematic representation of promoter (P*_fsptA_* or P*_tnaA_*)-preceded recombinant genes on expression plasmids is shown.

To investigate whether the putative VapBCs are functional or not, the eight putative VapC toxins were heterologously expressed in *Hfx. volcanii*, driven by a tryptophan-inducible promoter P*_tnaA_* (49). Similar to *Hfx. volcanii* containing the blank vector pSY1 (50), the strain expressing NJ7G_2730, NJ7G_3795, NJ7G_3855, or NJ7G_3857 grew well on the Hv-Cab (51) agar plate with tryptophan (Fig. 3A). By contrast, the strains harboring the expression plasmids for NJ7G_0346, NJ7G_0581, NJ7G_2438, and NJ7G_4368, respectively, displayed severe growth defects under the same conditions (Fig. 3A), suggesting that these four VapCs are toxic to *Hfx. volcanii* cells. When grown in Hv-Cab liquid medium, the strains expressing the four toxic VapCs also showed growth defects, as evidenced by their lower cell densities (Fig. 3B) and significantly lower viable cell numbers (Fig. 3C) compared with the control strain containing pSY1. Nevertheless, the strain expressing NJ7G_4368 displayed a better growth than those expressing NJ7G_0346, NJ7G_0581, or NJ7G_2438 (Fig. 3B), suggesting that the former exhibits a weaker toxicity than the latter three in *Hfx. volcanii*.

The functional relationship between toxin and its cognate antitoxin was investigated by co-expressing them in *Hfx. volcanii*, where the expression of VapC and VapB were driven by an inductive P*_tnaA_* and a constitutive promoter P*_fsptA_* (50), respectively (Fig. 3D). In contrast to the growth-defective strain harboring the expression plasmid only for the VapC toxin, the strain carrying the co-expression plasmid for NJ7G_0345/NJ7G_0346, NJ7G_0582/NJ7G_0581, NJ7G_2439/NJ7G_2438, or NJ7G_2321/NJ7G_4368 was able to grow on the Hv-Cab agar plate with tryptophan (Fig. 3D). These results suggest that NJ7G_0345, NJ7G_0582, NJ7G_2439, and NJ7G_2321 could act as VapB antitoxins to neutralize their cognate VapC toxins.

We next performed gene deletion analysis of the VapBCs in strain J7-2 using the endogenous CRISPR-Cas system-based genome-editing method (52). The single gene-deletion mutants for the antitoxins NJ7G_2729, NJ7G_3796, NJ7G_3854, and NJ7G_3856, whose cognate VapCs (NJ7G_2730, NJ7G_3795, NJ7G_3855, and NJ7G_3857) did not show toxicity to *Hfx. volcanii* (Fig. 3A), could be obtained (Fig. S2). However, we were unable to obtain single gene-deletion mutants for the antitoxins NJ7G_0345, NJ7G_0582, NJ7G_2439, and NJ7G_2321 (data not shown), whose cognate VapCs (NJ7G_0346, NJ7G_0581, NJ7G_2438, and NJ7G_4368) were toxic to *Hfx. volcanii* (Fig. 3A), implying that these four VapBs are essential for the survival of strain J7-2 by neutralizing their cognate VapC toxins. In support of this possibility, these four essential VapB genes and their cognate VapC genes could be deleted simultaneously to generate double gene-deletion mutants (Fig. S2).

The above-mentioned results demonstrate that NJ7G_0345/NJ7G_0346, NJ7G_0582/NJ7G_0581, NJ7G_2439/NJ7G_2438, and NJ7G_2321/NJ7G_4368 are functional in both *Hfx. volcanii* and strain J7-2. Based on the evidence that three of the four functional VapBCs of strain J7-2 possess an antitoxin with a RHH domain (NJ7G_0345, NJ7G_2439, and NJ7G_2321; Fig. 2B), we chose NJ7G_0345/NJ7G_0346 as a representative for further study. Hereafter, NJ7G_0345 and NJ7G_0346 were named NgVapB1 and NgVapC1, respectively, and the TA system constituted by them was named NgVapBC1.

### NgVapB1 neutralizes NgVapC1 by forming a complex

**Fig 4:**
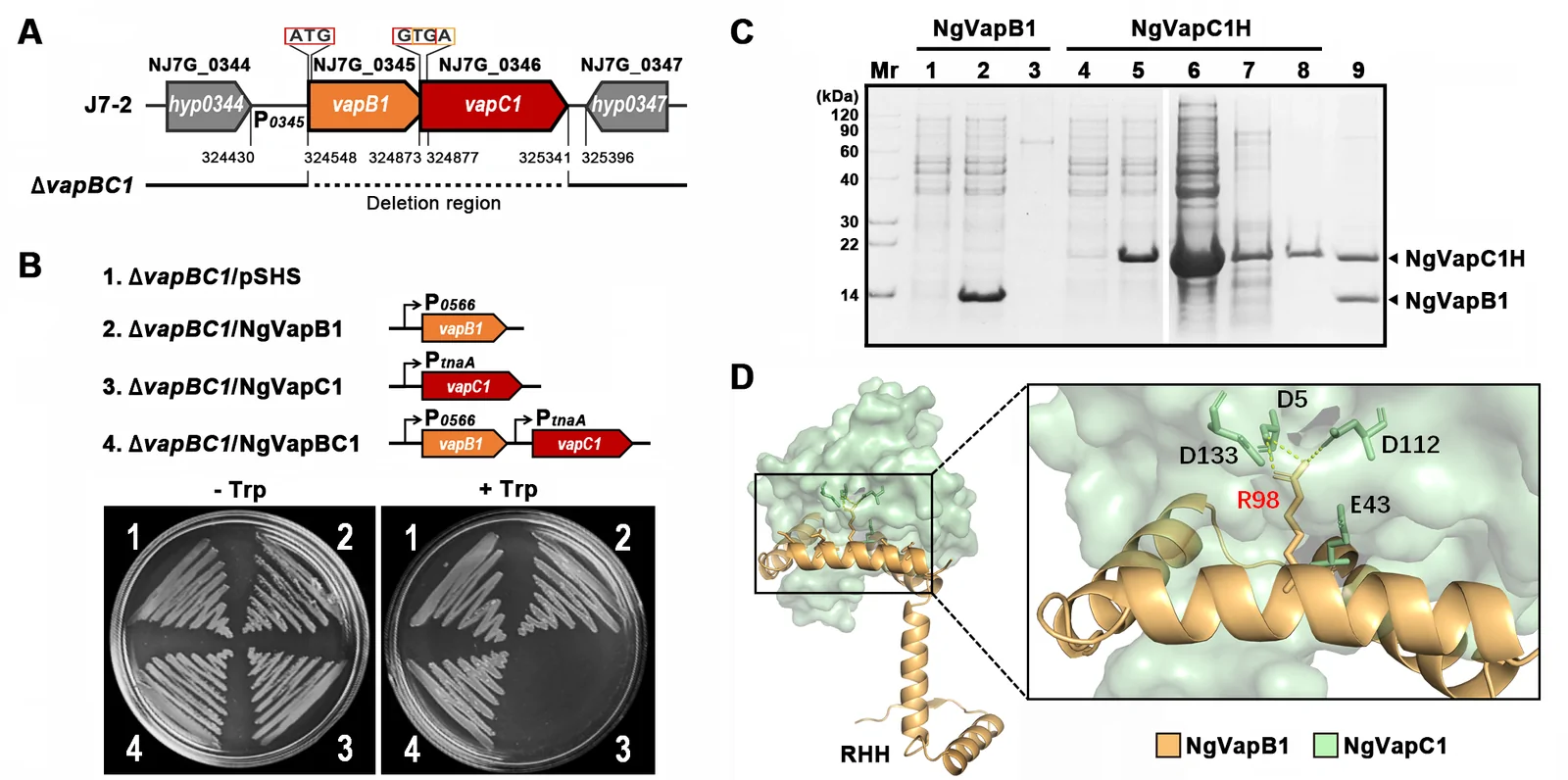
The interaction between NgVapB1 and NgVapC1. (**A**) Schematic view of *vapBC1* operon and adjacent genes in strain J7-2 and the deletion region in the mutant Δ*vapBC1*. (**B**) *In vivo* interaction between NgVapC1 and NgVapB1. Δ*vapBC1* strain carrying the blank vector (pSHS) or the expression plasmid for NgVapB1, NgVapC1, or both NgVapB1 and NgVapC1 was streaked on mevinolin (5 μg/mL)-containing 18% Hv-Cab agar plate without (−) or with (+) 1 mM tryptophan (Trp) and grown at 37°C for 4 days. Schematic representation of promoter (P*_0566_* or P*_tnaA_*)-preceded recombinant genes on expression plasmids is shown. (**C**) *In vitro* pull-down assay for the interaction between NgVapB1 and NgVapC1. The cellular proteins of *E. coli* harboring the expression plasmid for NgVapB1 or His-tagged NgVapC1H before (lanes 1 and 4) and after (lanes 2 and 5) IPTG induction, the inclusion bodies (lane 6) and refolded sample (lane 7) of NgVapC1H, as well as the eluted fraction from the nickel-affinity column for separation of the sample containing NgVapB1 (lane 3), NgVapC1H (lane 8), or both NgVapB1 and NgVapC1H (lane 9) were subjected to SDS-PAGE. (**D**) Predicted structural model of the NgVapBC1 complex. The four acidic residues in the active site (Asp5, Glu43, Asp112, and Asp133) of NgVapC1 and the residue Arg98 of NgVapB1 are shown. Dotted lines represent the ion pairs formed between the side chain of Arg98 and those of Asp5 and Asp112.

The double gene-deletion mutant Δ*vapBC1*, in which the coding regions for NgVapB1 and NgVapC1 were deleted (Fig. 4A and S2), was used as the host to investigate the interaction between NgVapB1 and NgVapC1. By using pSHS (52) as the vector, we constructed the expression plasmid for either or both NgVapB1 and NgVapC1, driven by a constitutive promoter (P*_0566_*) of a high-abundance blue copper domain protein (NJ7G_0566) of strain J7-2 (53) and inductive P*_tnaA_*, respectively (Fig. 4B). Similar to that in *Hfx. volcanii* (Fig. 3D), the expression of NgVapC1 alone in Δ*vapBC1* led to a severe growth defect, while the expression of NgVapB1 or co-expression of NgVapB1 and NgVapC1 did not affect the growth of the mutant strain (Fig. 4B), suggesting that NgVapB1 could neutralize the toxicity of NgVapC1 *in vivo*.

We further used an *in vitro* pull-down assay to confirm the interaction between NgVapB1 and NgVapC1. After expression in *E. coli*, recombinant NgVapC1 with a C-terminal His-tag (NgVapC1H) existed in inclusion bodies, which could be converted into soluble form after refolding and then purified by Ni-NTA affinity chromatography (Fig. 4C, lane 8). Untagged NgVapB1 was produced in *E. coli* as soluble form, which alone failed to bind the Ni-NTA resin (Fig. 4C, lane 3) but could be co-eluted with NgVapC1H from the column with a molar ratio of approximately 1:1 by band intensity quantification (Fig. 4C, lane 9), suggesting that the two proteins can interact with each other to form a stable NgVapBC1 complex. The structural prediction of this complex by AlphaFold 3 reveals that the three C-terminal α-helices of NgVapB1 bind to NgVapC1 and the side chain of Arg98 from NgVapB1 electrostatically interacts with those of Asp5 and Asp112 in the active site of NgVapC1 to inhibit the activity of the toxin (Fig. 4D), which is a common interaction mode conserved in VapBC complexes (9).

### A pseudo-palindromic sequence (PPS) in the *vapBC1* operon promoter functions as a negative *cis*-regulatory element for *vapBC1* expression

**Fig 5:**
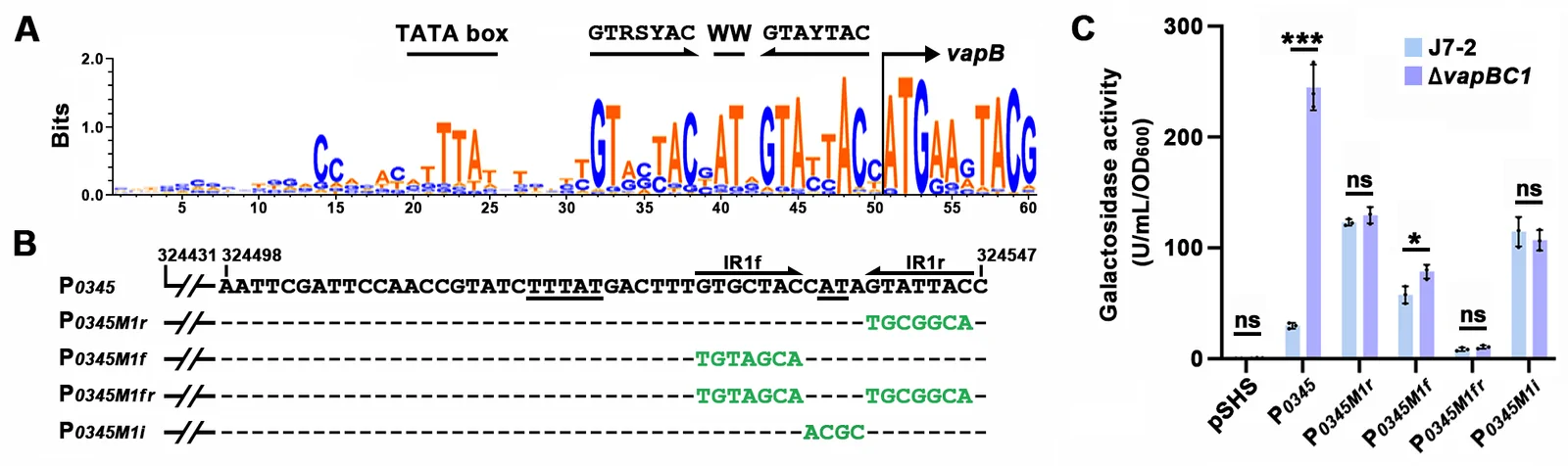
The effect of PPS1 on the *vapBC1* operon promoter activity. (**A**) The sequence logo of promoter regions of NgVapBC1 and its 31 homologs from haloarchaea. The conserved inverted repeat motifs of PPS1 are indicated by arrows. The TATA box and WW motif are indicated. (**B**) Sequences of the NgVapBC1 promoter region (P*_0345_*) and its mutants. The mutated and unaltered nucleotides are shown in letters and dashes, respectively. The inverted repeats (IR1f and IR1r) are indicated by arrows. The TATA box and WW motif are underlined. (**C**) Promoter activity assay. Strain J7-2 and Δ*vapBC1* carrying the blank vector (pSHS) or the expression plasmid for BgaH, driven by the indicated promoter, were grown in 23% MGM at 37°C to mid-exponential phase, followed by BgaH activity assay of cell extracts. The data are expressed as means ± SDs from biological triplicates (***, *P* < 0.001; **, *P* < 0.01; ns, no significance).

It has been reported that VapB antitoxins and VapBC complexes could bind to pseudo-palindromic, inverted repeats in their own operon promoters and negatively autoregulate their own transcription (9, 13). In addition to the highly conserved *cis*-acting elements (TATA box and WW motif) of haloarchaeal promoters (54), the promoter (P*_0345_*) region of *vapBC1* operon of strain J7-2 also contains a PPS (GTGCTAC-N4-GTATTAC; PPS1) comprising a pair of inverted repeats (IR1f/IR1r), which flank the WW motif and are well conserved in promoter regions of *vapBC1* operon homologs from 31 haloarchaea (Fig. 5A and S3). We used electrophoretic mobility shift assays (EMSAs) to investigate whether PPS1 can bind NgVapB1 or the NgVapBC1 complex *in vitro*. Recombinant NgVapB1 with a C-terminal His-tag (NgVapB1H) was produced in *E. coli* in a soluble form and purified (Fig. S4A). The DNA fragment covering the region of P*_0345_* (117 bp) and its mutated fragments, including P*_0345M1r_*, P*_0345M1f_*, and P*_0345M1fr_* with mutation of either or both IR1f and IR1r and P*_0345M1i_* with mutation of the 4-bp spacer between them (Fig. 5B), were used for EMSA analysis. The increase in NgVapB1H concentration led to a band shift for P*_0345_* (Fig. S4B), indicating that this antitoxin could bind to its own promoter. In the presence of 0.1 µM NgVapB1H, a band shift was observed for P*_0345_* but not for P*_0345M1r_*, P*_0345M1f_*, or P*_0345M1fr_*; at 0.2 µM NgVapB1H, P*_0345_* and P*_0345M1f_*, as well as P*_0345M1i_*, exhibited pronounced band shifts, while P*_0345M1r_* and P*_0345M1fr_* only showed a weak band shift; when NgVapB1H concentration increased to 0.4 µM, P*_0345M1r_* and P*_0345M1fr_* also exhibited pronounced band shifts as P*_0345_* and P*_0345M1f_* did (Fig. S4C). These results reflect the important role of PPS1, particularly IR1r, in the binding of NgVapB1. Our attempts to probe the interaction between the NgVapBC1 complex and its native promoter via EMSA failed because the purified NgVapBC1 complex (Fig. 4C, lane 9) tended to form aggregates (data not shown), similar to the case of the FitAB complex (10).

We then investigated the role of PPS1 in controlling *vapBC1* expression *in vivo*, with the halophilic β-galactosidase (BgaH)-encoding *bgaH* (55) as a reporter gene. When grown in modified growth medium containing 3.1 M NaCl (23% MGM), strain J7-2 harboring the *bgaH* expression plasmid driven by P*_0345_* exhibited much lower BgaH activity than Δ*vapBC1* carrying the same plasmid (Fig. 5C), indicating that NgVapB1 and NgVapC1 repress the promoter activity of P*_0345_* in strain J7-2. By contrast, there was no significant difference in BgaH activity between strain J7-2 harboring the *bgaH* expression plasmid driven by P*_0345M1r_* or P*_0345M1fr_* and Δ*vapBC1* carrying the same plasmid; meanwhile, the difference in BgaH activity between strain J7-2 carrying the *bgaH* expression plasmid driven by P*_0345M1f_* and Δ*vapBC1* carrying the same plasmid is much less significant than that driven by P*_0345_* (Fig. 5C). These results suggest that PPS1 acts as a negative *cis*-regulatory element and its mutation could relieve the repression effect of NgVapB1 and NgVapC1 on the promoter activity. We noticed that there was also no significant difference between strain J7-2 and Δ*vapBC1* in expression level of BgaH driven by P*_0345M1i_* (Fig. 5C), implying the spacer between IR1f and IR1r is involved in the function of PPS1. In Δ*vapBC1* that lacks NgVapB1 and NgVapC1, the expression level of BgaH driven by the mutant (P*_0345M1r_*, P*_0345M1f_*, P*_0345M1fr_*, or P*_0345M1i_*) was lower than that driven by P*_0345_* (Fig. 5C), suggesting that the mutation of PPS1 or the WW motif affects the promoter activity.

### The NgVapBC1 complex is a stronger repressor than NgVapB1 for autoregulation of the *vapBC1* operon

**Fig 6:**
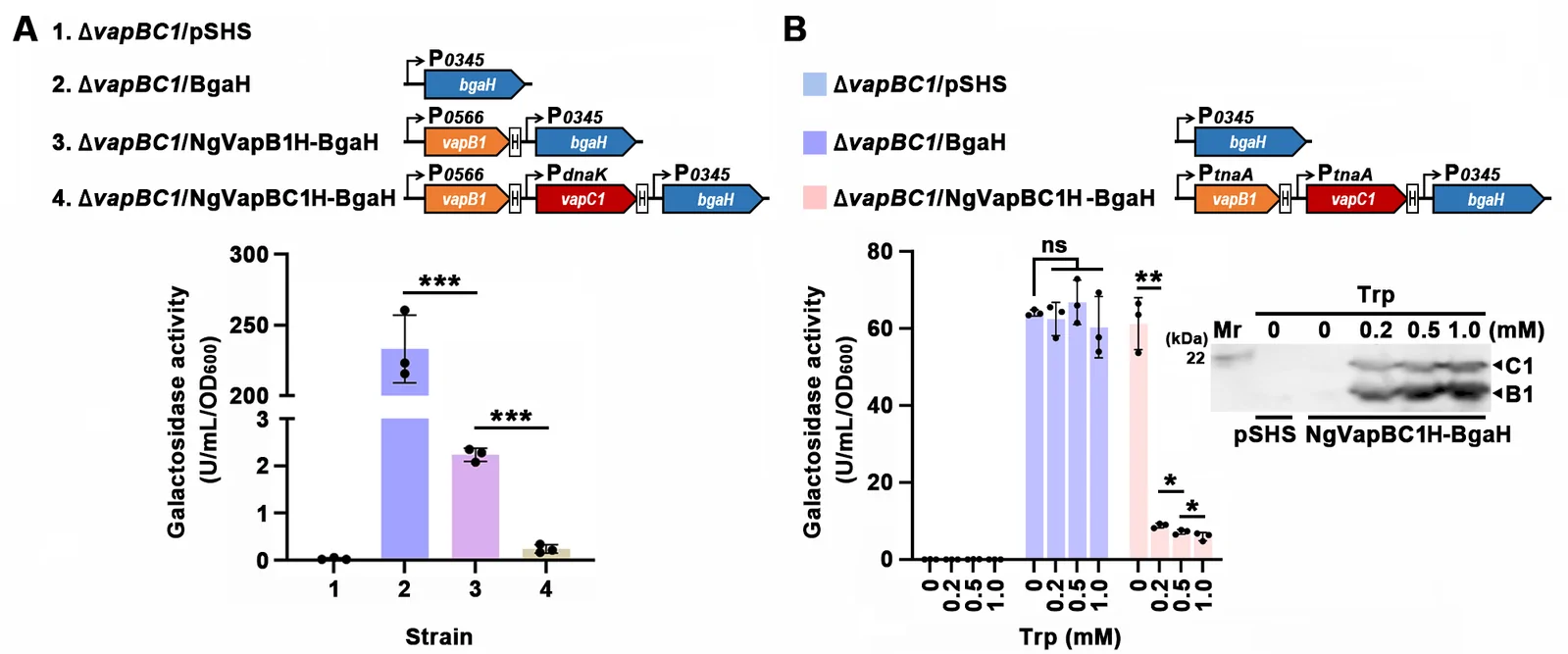
Comparison of the capacities between NgVapB1 and NgVapBC1 complex to repress P*_0345_* promoter activity (**A**) and dose-dependent effect of NgVapBC1 complex on P*_0345_* promoter activity (**B**). Δ*vapBC1* strains carrying the blank vector (pSHS) or the expression plasmid for BgaH alone or along with NgVapB1H or both NgVapB1H and NgVapC1H were grown at 37°C to mid-exponential phase in 23% MGM (**A**) or in 18% Hv-Cab with different concentrations of tryptophan (Trp) (**B**), followed by BgaH activity assay and anti-His-tag immunoblot analysis (B, inset) of cell extracts. The arrowheads indicate the positions of NgVapC1H (C1) and NgVapB1H (B1). Schematic representation of promoter (P*_0345_*, P*_0566_*, P*_dnaK_*, or P*_tnaA_*)-preceded recombinant genes on expression plasmids is shown. The data are expressed as means ± SDs from biological triplicates (***, *P* < 0.001; **, *P* < 0.01; *, *P* < 0.05; ns, no significance).

To investigate whether NgVapB1 and NgVapBC1 complex differ from each other in their capacities to repress *vapBC1* expression, we constructed the plasmid for co-expression of P*_0345_*-driven BgaH with NgVapB1H or with both NgVapB1H and C-terminal His-tagged NgVapC1H (Fig. 6A). In an effort to ensure an excess of antitoxin over toxin to prevent possible toxic effect of NgVapC1 on host cells, the strong constitutive promoter P*_0566_* and a relatively weak *dnaK* promoter (P*_dnaK_*) of *Hfx. volcanii* (Fig. S5) were employed to drive the expression of NgVapB1H and NgVapC1H, respectively (Fig. 6A). The Δ*vapBC1* strain expressing BgaH alone exhibited much higher BgaH activity than the strain co-expressing BgaH and NgVapB1H, while the strain co-expressing BgaH, NgVapB1H, and NgVapC1H showed almost no BgaH activity (Fig. 6A). These results suggest that NgVapB1 could repress the promoter activity of the *vapBC1* operon and NgVapBC1 complex has a higher repression activity than NgVapB1 alone.

When P*_0345_*-driven BgaH was co-expressed with tryptophan-inducible P*_tnaA_*-driven NgVapB1H and NgVapC1H in Δ*vapBC1*, the measured BgaH activity decreased with the increase of inducer concentration, accompanied by the increase in the amounts of NgVapB1H and NgVapC1H as evidenced by anti-His tag immunoblot analysis (Fig. 6B). By contrast, the BgaH activity exhibited by the strain expressing P*_0345_*-driven BgaH alone was not affected by the change of tryptophan concentration (Fig. 6B). These results imply that the expression level of NgVapBC1 complex positively correlates with its ability to repress *vapBC1* operon promoter *in vivo*.

### NgVapB1 is more resistant to proteolytic degradation when co-expressed with NgVapC1

**Fig 7:**
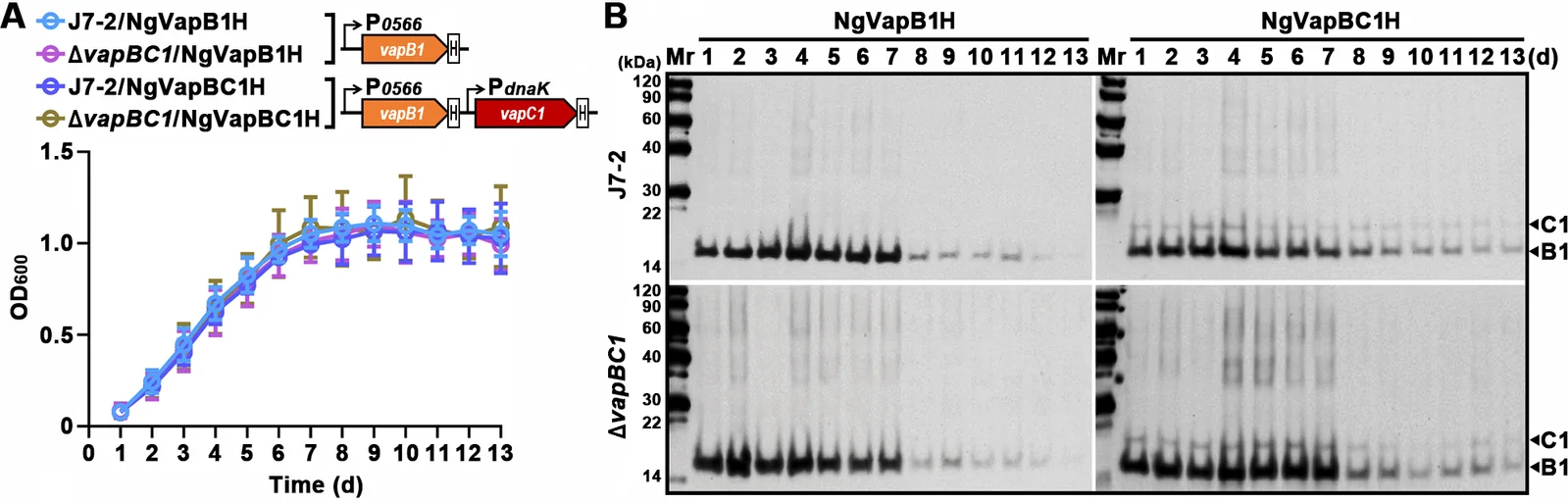
*In vivo* proteolytic degradation of NgVapB1. The target strains were grown in 23% MGM at 37°C. At the indicated time points, samples were taken and subjected to OD_600_ measurement (**A**) and anti-His-tag immunoblot analysis (**B**). Schematic representation of promoter (P*_0566_* or P*_dnaK_*)-preceded recombinant genes on expression plasmids are shown, and the data are expressed as means ± SDs from biological triplicates (**A**). The arrowheads indicate the positions of NgVapC1H (C1) and NgVapB1H (B1) (**B**).

The expression plasmid for NgVapB1H alone or the co-expression plasmid for both NgVapB1H and NgVapC1H was transferred into strain J7-2 or Δ*vapBC1*, and the resulting strains showed similar growth curves when grown in 23% MGM (Fig. 7A). Immunoblot analysis revealed that when the strains entered the stationary phase (8 days), NgVapB1H suffered severe proteolytic degradation by unidentified cellular protease(s) (Fig. 7B). We noticed that after 13-day cultivation, NgVapB1H molecules were almost completely degraded in strain J7-2 or Δ*vapBC1* when expressed alone but were partially retained when co-expressed with NgVapC1H (Fig. 7B), implying that the formation of NgVapBC1 complex endows the antitoxin with increased resistance to proteolysis.

It was previously reported that overexpression of Lon protease in *E. coli* leads to lethality, partly because the degradation of the antitoxin of a type II TA system (YefM-YoeB) by this protease liberates the toxin activity (56). Accordingly, we overexpressed strain J7-2 LonB protease (NgLonB) with a C-terminal His-tag (NgLonBH) in this haloarchaeon, driven by promoter P*_sptA_* (50). However, the overexpression strain (J7-2/NgLonBH) and the control strain (J7-2/pSHS) showed similar growth curves (Fig. S6), suggesting that active NgVapC1 has not been released. We noticed that NgLonBH suffered degradation over time (Fig. S6, inset), which is consistent with a previous report on the Lon protease of *Caulobacter crescentus* (57).

### Differential start codon usage by *vapB1* and *vapC1* and a −4 nt overlap of their stop and start codons contribute to the prevention of abnormal release of toxicity by the toxin

**Fig 8:**
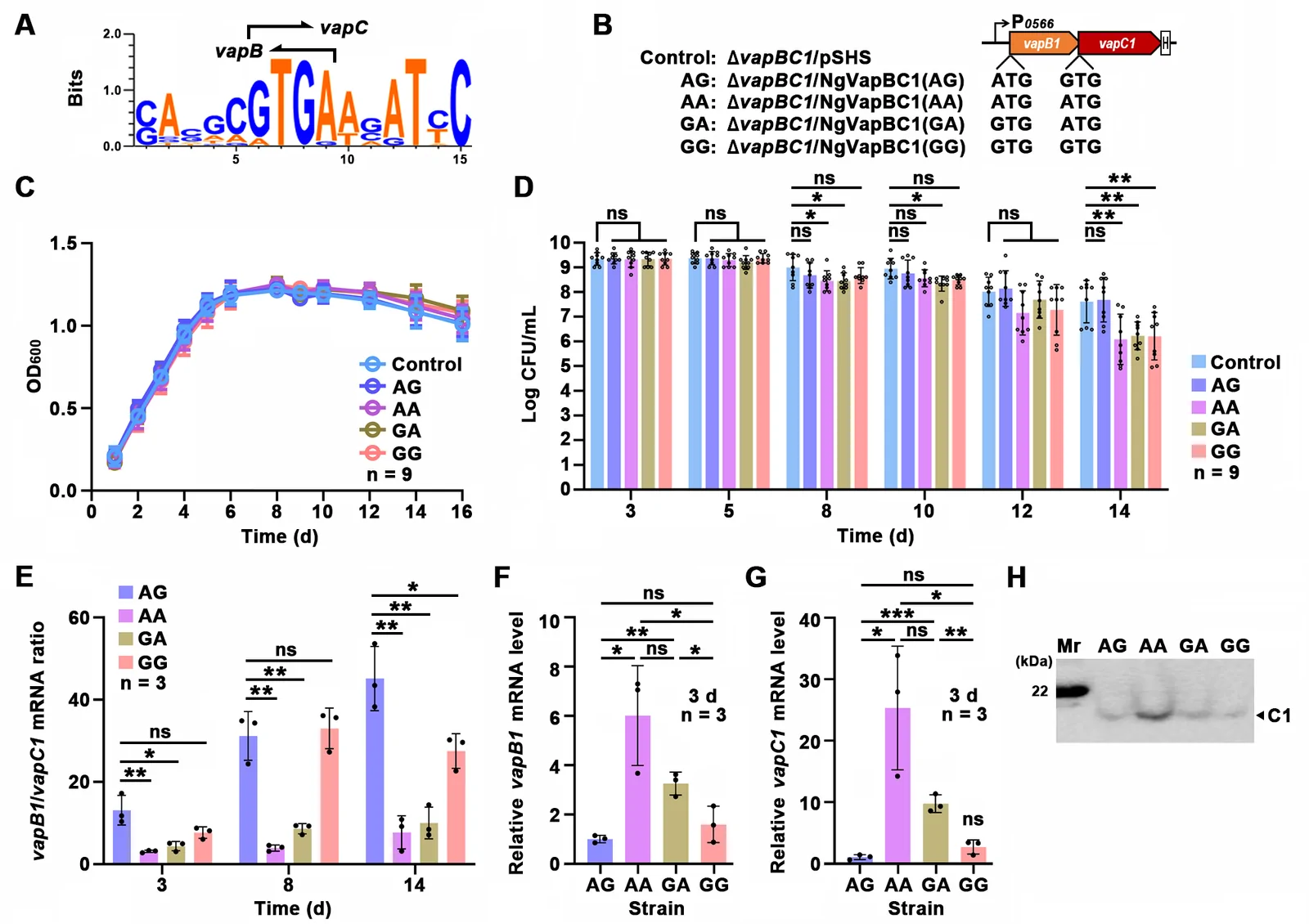
Role of start codon usage bias in *vapBC1* operon expression. (**A**) The sequence logo of the regions around the start codon of NgVapC1 and its 16 homologs from haloarchaea. (**B**) Schematic representation of P*_0566_*-preceded recombinant genes with different start codons in expression plasmids. (**C–H**) Effect of differential start codon usage on expression of NgVapB1 and NaVapC1. The target strains were grown in 23% MGM at 37°C. At the indicated time points, samples were taken and subjected to OD_600_ measurement (**C**), viable cell counting on 23% MGM agar plate at 37°C (**D**), determination of transcript levels by qRT-PCR analysis (E, F, and G), and anti-His-tag immunoblot analysis (**H**). For panel E, the *vapB1*/*vapC1* mRNA ratio was calculated directly using the measured transcript levels of *vapB1* and *vapC1*. For panels F and G, the values of the samples of AA, GA, and GG are relative to that of the sample of AG. For panel H, the arrowhead indicates the position of NgVapC1H (C1). The data are expressed as means ± SDs (***, *P* < 0.001; **, *P* < 0.01; *, *P* < 0.05; ns, no significance).

In the *vapBC1* operon, there is a −4 nt overlap (GTGA) of the *vapB1* stop codon (TGA) and the *vapC1* start codon (GTG), and this overlap motif is conserved in haloarchaeal *vapBC* operons homologous to *vapBC1* (Fig. 8A). Moreover, ATG and GTG are utilized as start codons by *vapB1* and *vapC1*, respectively (Fig. 4A), and this start codon usage bias is also conserved in haloarchaeal *vapB* (Fig. 5A) and *vapC* (Fig. 8A). To investigate the impact of the differential start codon usage and the −4 nt overlap motif on NgVapBC1 regulation, we constructed *vapBC1* operon mutants with different start codon usage patterns for *vapB1* and *vapC1* (Fig. 8B). When grown in 23% MGM, Δ*vapBC1* strains carrying the expression plasmid for wild-type *vapBC1* operon (strain AG) or its mutant (strain AA, GA, or GG) showed a similar growth curve to the control strain carrying pSHS by cell density quantification (Fig. 8C). Nevertheless, when entering the decline phase (e.g., 14 days), strains AA, GA, and GG showed significantly lower viable cell numbers than strain AG, while strain AG and the control strain displayed a similar viable cell number throughout the whole growth period (Fig. 8D). These results suggest that the use of ATG by *vapB1* and GTG by *vapC1* as start codons contributes to preventing the release of toxicity by NgVapC1.

Although *vapB1* and *vapC1* are organized as a bicistronic operon, the qRT-PCR analysis of strain AG revealed a *vapB1*/*vapC1* mRNA ratio of 13.1, 31.2, and 45.1 at exponential (3 days), stationary (8 days), and decline (14 days) phases, respectively (Fig. 8E), indicating that the *vapBC1* transcript suffers from truncation of *vapC1* mRNA. Under the same conditions, *vapB1*/*vapC1* mRNA ratios of strains AA and GA were lower than those of strains AG and GG (Fig. 8E). Compared with strain AG, strain AA showed higher mRNA levels of *vapB1* (Fig. 8F) and *vapC1* (Fig. 8G), as well as a higher level of NgVapC1H (Fig. 8H). These results demonstrate that the use of ATG and GTG as start codons by *vapB1* and *vapC1*, respectively, correlates with maintaining a high *vapB1*/*vapC1* mRNA ratio and a lower level of *vapC1* mRNA to avoid abnormal accumulation of excess toxin. Moreover, in each of the strains, the relative amounts of NgVapC1H (Fig. 8H) are roughly correlated with the relative levels of *vapC1* mRNAs (Fig. 8G), reflecting the importance of differential start codon usage by *vapB1* and *vapC1* in controlling the levels of transcripts to modulate the amounts of antitoxin and toxin. Notably, regardless of which start codon (ATG or GTG) was used by *vapB1*, the use of GTG rather than ATG by *vapC1* as the start codon led to lower mRNA levels of both *vapB1* and *vapC1* (Fig. 8F and G), suggesting that the start codon of the toxin is involved in the stability of the *vapBC1* transcript.

**Fig 9:**
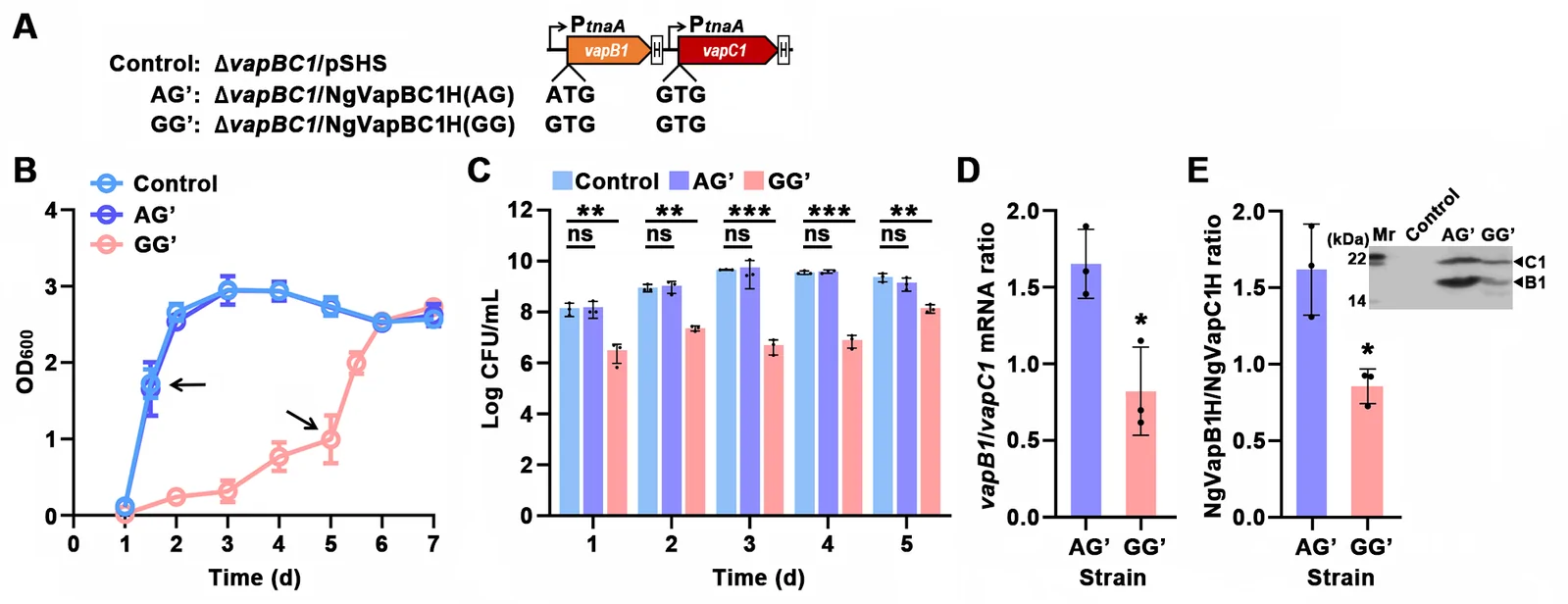
Effect of start codon on ratio of NgVapB1 to NaVapC1 synthesized by separate monocistronic mRNAs. (**A**) Schematic representation of P*_tnaA_*-preceded recombinant genes with different start codons in expression plasmids. (**B–E**) Effect of start codon on expression of NgVapB1 and NaVapC1. The target strains were grown at 37°C in 18% Hv-Cab supplemented with 1 mM tryptophan and 5 μg/mL mevinolin. At the indicated time points, samples were taken and subjected to OD_600_ measurement (**B**), viable cell counting on Hv-Cab agar plate at 37°C (**C**), determination of transcript levels by qRT-PCR analysis (**D**), and anti-His-tag immunoblot analysis (**E**). The arrows in panel B indicate the sampling time points (the exponential phase) for qRT-PCR analysis (**D**) and anti-His-tag immunoblot analysis (**E**). For panel E, the relative amounts of NgVapB1H and NgVapC1H were determined by quantifying the band density (inset), and the arrowheads indicate the positions of NgVapC1H (C1) and NgVapB1H (B1). The data are expressed as means ± SDs from biological triplicates (***, *P* < 0.001; **, *P* < 0.01; *, *P* < 0.05; ns, no significance).

We next investigated whether differential start codon usage influences the synthesis of NgVapB1 and NaVapC1 by separate monocistronic genes and probed the role of the −4 nt overlap in regulation of the NgVapBC1 system. The plasmid for co-expressing *vapB1* with ATG or GTG start codon and *vapC1* with GTG start codon, individually driven by P*_tnaA_*, was constructed and transferred into Δ*vapBC1*, yielding the strains AG’ and GG’ (Fig. 9A). When grown in Hv-Cab medium with mevinolin and tryptophan, strain AG’ displayed a similar growth curve and viable cell number to the control strain carrying pSHS throughout the cultivation period, while strain GG’ exhibited a remarkable growth defect (Fig. 9B and C). At the exponential phase, strain AG’ showed a higher *vapB1*/*vapC1* mRNA ratio (Fig. 9D) and a higher NgVapB1H/NaVapC1H ratio than strain GG’ (Fig. 9E), indicating that the replacement of the start codon ATG of *vapB1* by GTG decreases the relative amount of the antitoxin to promote the release of toxicity by the toxin.

We noticed that the *vapB1*/*vapC1* mRNA ratios of exponential-phase strains AG’ (1.7) and GG’ (0.8) expressing the separate monocistronic genes (Fig. 9D) were much lower than those of strains AG (13.1) and GG (7.7) expressing the bicistronic genes with the −4 nt overlap (3 days, Fig. 8E). These results suggest that the −4 nt overlap motif in the *vapBC1* operon plays an important role in maintaining a higher *vapB1*/*vapC1* mRNA ratio for prevention of the synthesis of excess toxin to release toxicity abnormally.

### The NgVapBC1 system contributes to population heterogeneity of the decline-phase strain J7-2 and facilitates the survival of some subpopulations under stress conditions

**Fig 10:**
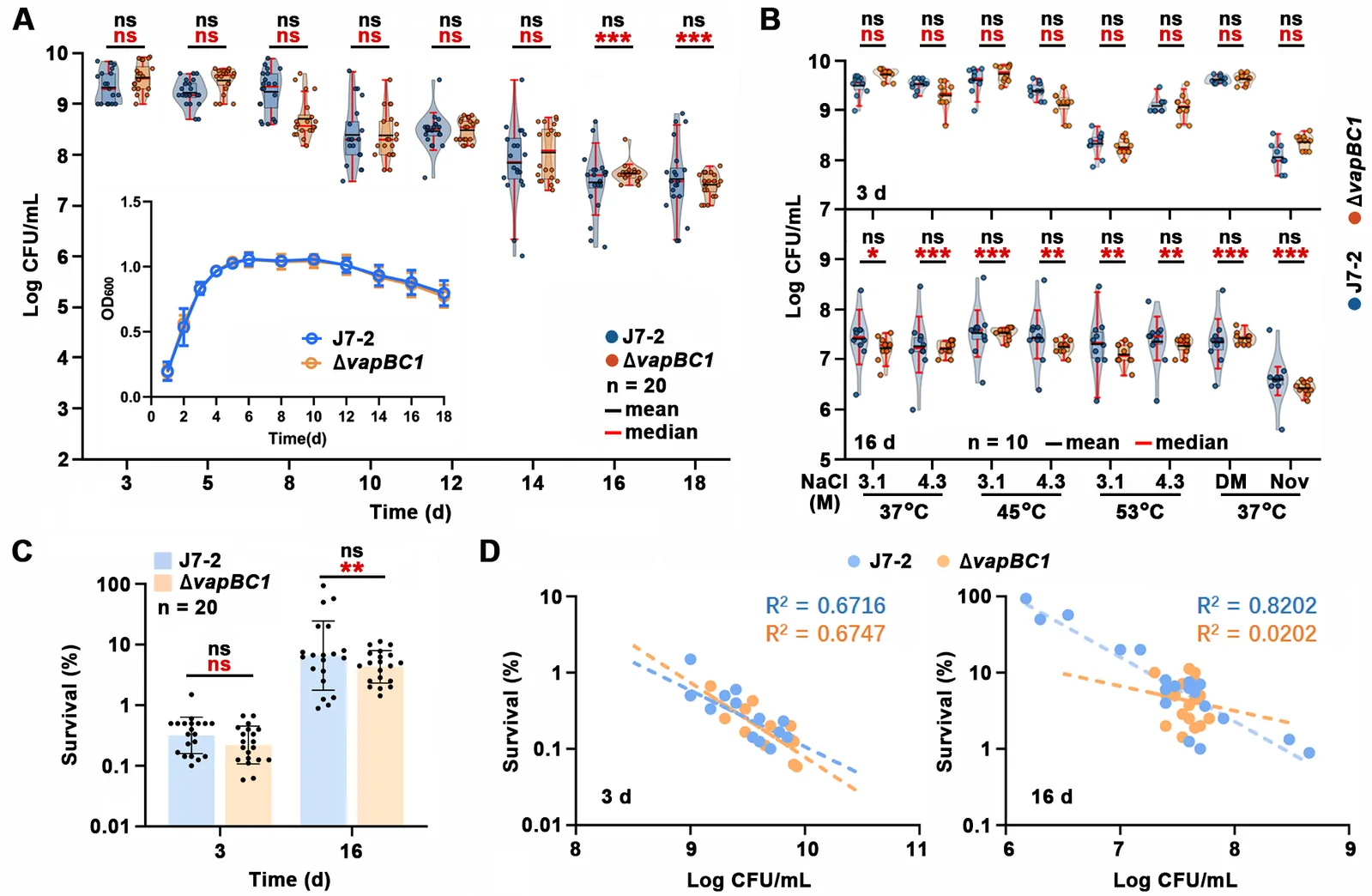
The role of NgVapBC1 system in growth and survival of Strain J7-2. Strain J7-2 and Δ*vapBC1* were grown in 23% MGM at 37°C, and samples were taken at the indicated time points for OD_600_ measurement (inset in panel A) and viable cell counting on 23% MGM agar plates at 37°C (**A**). The exponential (3 days) and decline (16 days) phase cells were serially diluted and spotted onto MGM agar plates with different NaCl concentrations, 5-fold diluted MGM agar plates with 3.1 M NaCl (DM), or 23% MGM agar plates with 0.4 μg/mL novobiocin (Nov) and then cultivated at the indicated temperatures for 7 days before viable cell counting (**B**). For UV sensitivity, serially diluted exponential (3 days) and decline (16 days) phase cells were spotted onto 23% MGM agar plates and exposed to UV at 80 J/m^2^, followed by cultivation at 37°C for 7 days in darkness before viable cell counting (**C**). The data in panels A and C were used for drawing correlation plots between the number of viable cells in the exponential (3 d) and decline (16 d) phase cultures and their survival after UV radiation (**D**). Significant differences between means (or medians) and variances of two groups are indicated in black and red, respectively (***, *P* < 0.001; **, *P* < 0.01; *, *P* < 0.05; ns, no significance).

To probe the physiological role of the NgVapBC1 system in strain J7-2, we compared the growth and survival of strain J7-2 and Δ*vapBC1* under different conditions. Although strain J7-2 and Δ*vapBC1* displayed similar growth curves by cell density quantification, the former showed a significantly higher variability among biological replicates in viable cell number than the latter at the decline phase (Fig. 10A; 16–18 days). The exponential (3 days) and decline (16 days) phase cells of strain J7-2 and Δ*vapBC1* were collected and subsequently cultivated at different temperatures (37°C–53°C) and salinities (3.1–4.3 M NaCl), under nutrient-limiting condition (DM), and in the presence of the antibiotic novobiocin, respectively (Fig. 10B). Compared with Δ*vapBC1*, the decline, rather than exponential-phase strain J7-2, still showed a higher variability among biological replicates in viable cell number under these conditions (Fig. 10B). These results suggest that the NgVapBC1 system contributes to population heterogeneity of the decline-phase strain J7-2.

The exponential- and decline-phase cells of strain J7-2 and Δ*vapBC1* were subjected to UV radiation. In contrast to the exponential-phase cells, the decline-phase strain J7-2 showed a higher variability among biological replicates in survival rate than the decline-phase Δ*vapBC1*, and some biological replicates of the decline-phase strain J7-2 displayed higher levels of survival rate than those of Δ*vapBC1* after UV exposure (Fig. 10C). These results suggest that the NgVapBC1 system-mediated population heterogeneity of strain J7-2 facilitates subpopulations to survive under stress conditions.

After UV exposure, the decline-phase cells of both strain J7-2 and Δ*vapBC1* displayed higher levels of survival rate than their exponential-phase cells (Fig. 10C); moreover, the numbers of viable cells in the exponential-phase cultures of strain J7-2 and Δ*vapBC1* are moderately and negatively correlated with their survival rates (R^2^ = ~0.67) after UV exposure (Fig. 10D), implying that less metabolically active cells are more resistant to UV stress. Notably, for the decline-phase cultures, the viable cell number of strain J7-2 is highly and negatively correlated with its survival rate (R^2^ = ~0.82) after UV exposure, whereas there is no correlation between the viable cell number and the survival rate (R^2^ = ~0.02) of Δ*vapBC1* under the same conditions (Fig. 10D). These results suggest that the NgVapBC1 system participates in the reduction of metabolic activity of decline-phase cells to improve their stress resistance.

**Fig 11:**
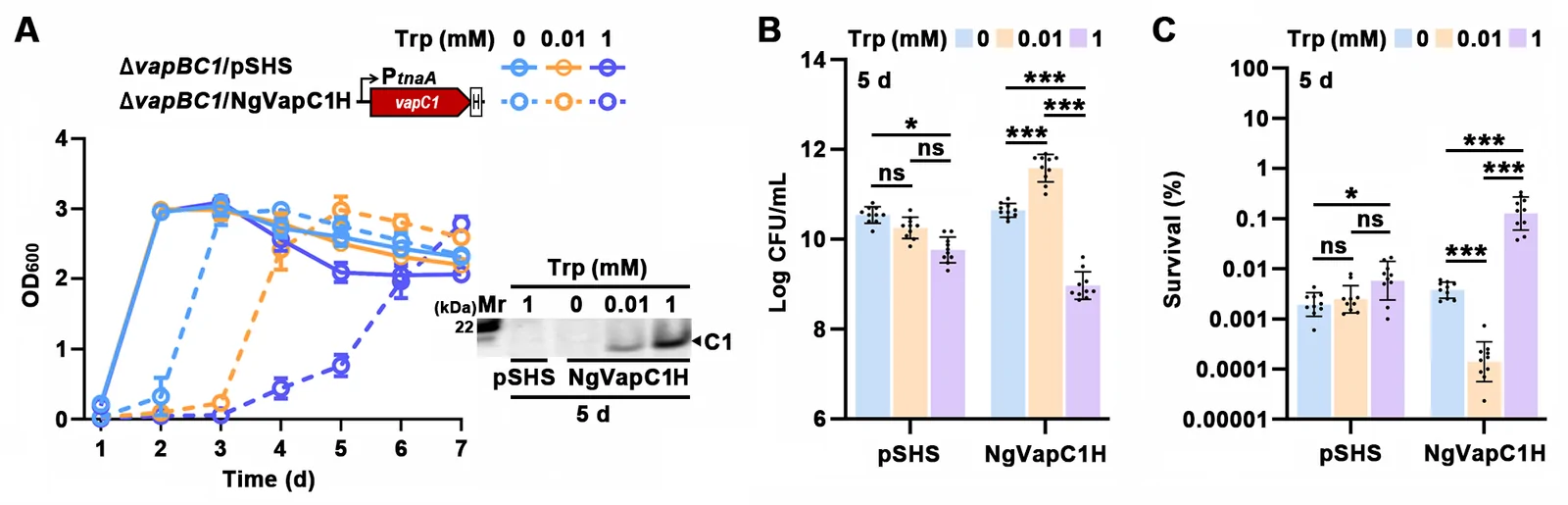
Effect of NgVapC1 on growth and survival of strain J7-2. The target strains were grown at 37°C in 18% Hv-Cab with 5 μg/mL mevinolin and different concentrations of tryptophan (Trp), and samples were taken at the indicated time points for OD_600_ measurement (**A**), anti-His-tag immunoblot analysis (A, inset; C1 indicates the position of NgVapC1H), and viable cell counting on Hv-Cab agar plates at 37°C (**B**). The cells of 5-day cultures were spotted onto Hv-Cab agar plates and exposed to UV at 80 J/m^2^, followed by cultivation at 37°C for 7 days in darkness before viable cell counting (**C**). The data are expressed as means ± SDs (*n* = 10; ***, *P* < 0.001; *, *P* < 0.05; ns, no significance).

We postulated that the population heterogeneity of decline-phase strain J7-2 may correlate with cell-to-cell fluctuation in active NgVapC1 level, which will lead to variation in metabolic activity and stress resistance of different subpopulations. To test this possibility, we employed the tryptophan-inducible promoter P*_tnaA_* to drive the expression of NgVapC1H in Δ*vapBC1* (Fig. 11A). With the increase in tryptophan concentration, the growth of Δ*vapBC1* harboring the expression plasmid for NgVapC1H (Δ*vapBC1*/NgVapC1H) was inhibited to a greater extent and the amount of cellular NgVapC1H was increased (Fig. 11A). In the absence of tryptophan, Δ*vapBC1*/NgVapC1H still showed a delay in growth compared with the control strain Δ*vapBC1*/pSHS (Fig. 11A), probably due to leaky expression from the P*_tnaA_* promoter. These results reflect the toxic effect of NgVapC1H on the metabolic activity of the cells. The 5-day cultures containing different concentrations of tryptophan were subjected to viable cell counting and UV treatment. The results showed that the numbers of viable cells (Fig. 11B) in the cultures are negatively correlated with their survival rates after UV exposure (Fig. 11C), in particular Δ*vapBC1*/NgVapC1H. These results validate the role of NgVapC1 in improving the stress resistance of strain J7-2 by reducing cellular metabolic activity.

## DISCUSSION

Our results show that four of the eight putative VapBCs of *Nnm. gari* J7-2 are functional. The NgVapBC1 system was further characterized in terms of its regulation at multiple levels and role in the establishment of population heterogeneity of the haloarchaeon.

### Nonfunctional and functional VapBCs

For the four nonfunctional VapBCs of strain J7-2, all of their toxins except NJ7G_3795 possess the four active-site residues of VapC toxins, but none of them show detectable toxicity in our assays. Similarly, the overexpression of some *M. tuberculosis*-derived VapCs with an intact active site in *M. bovis* does not result in growth inhibition (58). The mechanism for the nontoxicity of these VapCs remains to be further elucidated. The NJ7G_2729-GFP fusion protein was found to be recruited to the FtsZ ring in *Natrinema* sp. J7, implying that NJ7G_2729 participates in cell division (42). Considering that NJ7G_2730 and NJ7G_2729 are organized in an operon, we postulate that NJ7G_2730 may be involved in cell division. Additionally, the toxin MtvT of a plasmid-encoded type II TA system MtvTA exhibits no detectable toxicity, but the MtvTA system could regulate plasmid conjugative transfer and bacterial virulence in *Pseudomonas aeruginosa* (59). It is possible that in strain J7-2, the putative VapCs without cell toxicity, instead of acting as toxins, may actually exert other functions.

Besides NgVapBC1, the other three putative VapBCs (NJ7G_0582/NJ7G_0581, NJ7G_2439/NJ7G_2438, and NJ7G_2321/NJ7G_4368) of strain J7-2 are experimentally confirmed to be functional in strain J7-2 and *Hfx. volcanii*. The evidence that NJ7G_4368 exhibits a weaker toxicity to *Hfx. volcanii* than NgVapC1, NJ7G_0581, or NJ7G_2438 may reflect a functional diversity among these VapBCs. Nevertheless, regulatory mechanisms and physiological roles of the three functional VapBCs, as well as the possible relationship among the multiple TA systems of strain J7-2, remain to be elucidated.

### Transcriptional regulation

The antitoxin NgVapB1 or the NgVapBC1 complex negatively autoregulates its own transcription by binding to the pseudo-palindromic sequence PPS1 in the *vapBC1* operon promoter of strain J7-2. PPS1 is downstream of the TATA box recognized by the TATA-binding protein (TBP) and flanks the WW motif that possibly interacts with the transcription factor B (TFB) and RNA polymerase (RNAP) in haloarchaea (54). Because the formation of the TBP-TFB-DNA complex is required for recruiting RNAP to form the preinitiation complex (PIC) (60), the occupation of PPS1 by NgVapB1 or the NgVapBC1 complex would affect PIC formation necessary for *vapBC1* operon transcription. The conservation of PPS1 in *vapBC* operon promoters of haloarchaea emphasizes the role of this negative *cis*-regulatory element in autoregulation of haloarchaeal VapBC systems. To the best of our knowledge, the present results are the first evidence of the PPS-mediated autoregulation for archaeal type II TA systems. In bacteria, *vapBC* operons also employ PPSs for autoregulation (9, 13). While haloarchaeal *vapBC* operon PPSs are downstream of the TATA box, bacterial *vapBC* operon PPSs generally cover the −10 and/or −35 region, which can be occupied by the VapBC complex to prevent the interaction of the core promoter elements with RNAP holoenzyme (4, 61, 62). Despite the difference in the position of PPS to the TATA box in archaea and −35/−10 regions in bacteria, both archaeal and bacterial *vapBC* operons employ PPSs for binding toxin-antitoxin complexes to block the initiation of transcription, suggesting that the transcriptional autoregulation mechanism of VapBC systems is conserved across the two domains of life.

### Post-transcriptional and translational regulation

Strain J7-2 maintains a high *vapB1*/*vapC1* mRNA ratio through truncation of the *vapBC1* transcript within the toxin-coding region, allowing the production of an excess of NgVapB1 to prevent abnormal release of toxicity by NgVapC1. Transcripts with truncation of the toxin-coding region have been reported for bacterial type II TA systems. In the case of the MazEF system of *E. coli*, the toxin MazF cleaves the *mazEF* transcript specifically at ACA sites, leading to a high *mazE*/*mazF* mRNA ratio due to the presence of more ACA sites in the coding region of MazF (6). For the Kis-Kid system of plasmid R1, its truncated transcript originates from limited degradation of the toxin Kid-coding sequence containing a stem-loop structure, which is located in the 5′ region of *kid* and may act as a protective barrier against degradation of upstream mRNA by 3′–5′ exoribonucleases (5). Additionally, the RNA-seq data reveal toxin-coding region truncated transcripts of the DinJ-YafQ and YafNO systems of *E. coli*, but the underlying mechanism remains unclear (63). In strain J7-2, a single-base mutation (GTG → ATG) only in the start codon of *vapC1* causes a significant change in the *vapB1*/*vapC1* mRNA ratio, suggesting that the NgVapBC1 system is unlikely to maintain a higher *vapB1* mRNA level through a mechanism similar to MazEF (6) or Kis-Kid (5).

The −4 nt overlap (GTGA) of the *vapB1* stop codon and the *vapC1* start codon in the *vapBC1* operon is conserved in homologous *vapBC* operons of haloarchaea. It was recently reported that a −4 nt overlap (ATGA or GTGA) of the stop and start codons is the most common spacing motif in unidirectional gene pairs of prokaryotes and is required for translational coupling via termination-reinitiation (TeRe), whereby the same ribosome (or at least the small subunit) that terminates translation of the upstream gene reinitiates translation of the downstream gene that could not be expressed in the absence of translational coupling (64, 65). We found that separate monocistronic *vapB1* and *vapC1* generate a much lower *vapB1*/*vapC1* mRNA ratio than the bicistronic *vapBC1* operon, emphasizing the importance of translational coupling for modulating the antitoxin/toxin mRNA ratio. Moreover, the replacement of the start codon GTG of *vapC1* by ATG does not disrupt the integrity of the −4 nt overlap (GTGA → ATGA) but could improve the *vapBC1* transcript stability and decrease the *vapB1*/*vapC1* mRNA ratio. Consistent with this, ATG is more efficient than GTG not only in initiating translation but also in binding the ribosome to protect the transcript against RNase-mediated degradation in haloarchaea (50, 66). Given that the *vapBC1* operon employs the TeRe mechanism for translation, the truncated *vapBC1* transcript probably results from a weaker protective effect of the ribosome on the downstream toxin-coding region. Specifically, the translation of *vapC1* depends on the completion of the translation of upstream *vapB1*, and during translation, the polysome-associated *vapB1* mRNA is resistant to RNase; by contrast, the use of a “weak” start codon GTG by *vapC1* leads to less efficient translation, and thus, the *vapC1* mRNA is less occupied by ribosomes and more sensitive to RNase-mediated degradation. Moreover, translational coupling could facilitate the coordinated translational regulation of overlapping gene pairs to ensure a precise stoichiometry of interacting proteins (64, 65). In this context, the −4 nt overlap-mediated translational coupling of *vapB1* and *vapC1* allows both post-transcriptional and translational regulation of the NgVapBC1 system.

### Post-translational regulation

It has long been held that type II antitoxins are more sensitive to proteolysis than their cognate toxins, promoting the liberation of the latter (1, 56). However, accumulating evidence suggests that the activation of toxins in TA complexes is unlikely due to preferential degradation of the antitoxin because antitoxins are protected from degradation after forming stable TA complexes with toxins (1, 2, 7). In agreement with this, NgVapB1 could form a complex with NaVapC1, and it suffers severe proteolytic degradation when expressed alone in strain J7-2 but becomes more resistant to proteolysis when co-expressed with the toxin. Moreover, the evidence that overexpression of LonB protease in strain J7-2 does not result in lethality raises the possibility that active NgVapC1 could not be liberated through proteolytic degradation of NgVapB1 by LonB, in contrast to the *E. coli* YefM-YoeB system that is activated via Lon-dependent degradation of the antitoxin (56). Notably, haloarchaeal LonB proteases differ from their bacterial homologs in that the former are membrane-associated while the latter are typically soluble (67). It is presently unclear whether membrane association of LonB limits its action on NgVapB1. Additionally, we cannot rule out the possibility that NgVapB1 is inherently resistant to LonB. Some non-proteolytic mechanisms have been proposed for toxin activation of type II TA systems of *M. tuberculosis*, such as chaperone-mediated conformational change in HigBA complex (68), thermal remodeling of ParDE1 complexes (69), and phosphorylation of VapB to inhibit the formation of VapBC complex (70). Further studies are needed to investigate whether these non-proteolytic mechanisms are applicable to the NgVapBC1 system.

### Physiological roles

It was reported that the deletion of *vapBC6* in *S. solfataricus* or *vapBC4* in *S. acidocaldarius* leads to increased thermal sensitivity, indicating critical involvement of VapBCs in response to thermal stress in thermoacidophilic archaea (11, 12). By contrast, the exponential-phase cells of strain J7-2 and Δ*vapBC1* do not display phenotypic differences under different stress conditions, implying that NgVapBC1 does not function as a *bona fide* stress-responsive system. Instead, NgVapBC1 contributes to the population heterogeneity of the decline-phase strain J7-2. The establishment of heterogeneous populations with distinct toxin activities and metabolic capacities has been observed for several type II TA systems of bacteria (71). TA systems are tightly regulated at multiple levels and implemented in regulatory networks, of which all components can be differentially regulated at all levels to facilitate or hinder phenotypic switches via toxin activation (71, 72). When strain J7-2 cells enter the decline phase, the loss of cellular homeostasis would disturb the regulatory network of the NgVapBC1 system to trigger stochastic activation of NgVapC1, leading to cell-to-cell variation in toxin activity and metabolic capacity.

Our results suggest that the establishment of population heterogeneity of strain J7-2 is associated with NgVapBC1 and could confer fitness advantages to the haloarchaeon. Previous reports show that VapC toxins cleave RNAs (tRNA, rRNA, and/or mRNA) to inhibit translation, thereby slowing down cellular metabolism and growth (10–12). In general, slow-growing cells are more stress-tolerant than fast-growing ones (73). Indeed, the growth rate of strain J7-2 is negatively correlated with the survival rate of this haloarchaeon under UV stress. For the decline-phase strain J7-2, some subpopulations with a higher level of active NgVapC1 would grow more slowly but survive better under stress conditions; in contrast, other subpopulations with a lower level of active NgVapC1 are less stress-resistant but may contribute more to population restoration once the stress is relieved. Meanwhile, we do not exclude the possibility that the toxin-mediated inhibition of translation of certain proteins (e.g., DNA replication-related proteins) may affect the genome stability of strain J7-2 to generate genetic heterogeneity, which also enables the population to better withstand different stresses. In this context, the NgVapBC1-mediated growth and metabolic heterogeneity facilitate the survival and multiplication of the strain J7-2 population as a whole in diverse and changing environments. Given that VapC toxins act on RNAs, further single-cell transcriptomics analysis is expected to reveal the RNA substrates and the underlying mechanism responsible for NgVapBC1-mediated population heterogeneity of strain J7-2.

## MATERIALS AND METHODS

### Strains and growth conditions

The bacterial and haloarchaeal strains used in this study are listed in Table S1 in the supplemental material. Unless otherwise indicated, *Nnm. gari* J7-2 and its derivatives were grown aerobically at 37°C with shaking at 185 rpm in 23% MGM supplemented with 5 µg/mL mevinolin when necessary, as described previously (39). The Hv-Cab medium (51) was used for cultivating *Hfx. volcanii* WFD11, strain J7-2, and its derivatives expressing the target gene driven by the tryptophan-inducible promoter P*_tnaA_* at 37°C in the presence of the indicated concentration of tryptophan. When necessary, novobiocin (4 μg/mL) was added into the Hv-Cab medium. *E. coli* DH5a, *E. coli* JM110, and *E. coli* BL21(DE3) were used as hosts for plasmid construction, demethylation, and expression, respectively, and were grown at 37°C in Luria-Bertani medium supplemented with kanamycin (30 µg/mL) as needed. Solid medium was prepared by adding 1.5% (wt/vol) agar.

### Plasmid construction and mutagenesis

The plasmids and primers used in this study are listed in Tables S2 and S3, respectively. The *E. coli* expression plasmids were constructed by inserting the target gene fragment, amplified from the strain J7-2 genome by PCR, into the NdeI-NcoI site of pET26b (Novagen). The plasmids for genome editing of strain J7-2 were constructed as described previously (52). Briefly, a mini-CRISPR preceded by promoter P*_sptA_* (50) was synthesized by PCR using partially overlapping primers (Table S3). The homologous arms flanking the target gene were amplified from the strain J7-2 genome and connected by overlapping PCR to generate a donor DNA. Using the GenRec Assembly Master Mix Kit (generalbiol, Anhui, China), the P*_sptA_*-preceded mini-CRISPR and the donor DNA were inserted into the BamHI-XbaI site of pSHS, yielding the genome-editing plasmid. To construct the vectors for expressing recombinant proteins in haloarchaea (*Hfx. volcanii* WFD11, strain J7-2, and its derivatives), different promoters were amplified from the genome of strain WFD11 (P*_dnaK_* and *P_tnaA_*) or strain J7-2 (P*_sptA_*, P*_fsptA_*, *P_0566_*, and *P_0345_*), and connected with the target gene fragment amplified from the strain J7-2 genome or *bgaH* amplified from plasmid pST1 (50) by overlapping PCR, followed by insertion into the KpnI-NcoI site of pSY1 (50) or the BamHI-XbaI site of pSHS (52). The sequences of all recombinant plasmids were verified by DNA sequencing.

### Construction of gene-deletion mutants of *Nnm. gari* J7-2

Deletion of target genes in strain J7-2 was carried out using the endogenous CRISPR-Cas system-based genome-editing method as described previously (52) with minor modifications. Briefly, a non-methylated genome-editing plasmid was isolated from *E. coli* JM110 and then transferred into strain J7-2 through polyethylene glycol-mediated transformation, followed by plating on 18% MGM agar plates. For screening of the target mutant, randomly selected colonies were cultivated in liquid 23% MGM, and the cells were recovered by centrifugation and lysed with sterile deionized water, followed by PCR analysis with external and internal primer pairs specific for the target gene. For curing of the plasmid, the PCR-verified mutant was subjected to 2–3 rounds of subculturing (2 days for each round) in liquid 23% MGM, and the absence of the plasmid was confirmed by PCR analysis. Finally, the mutants were validated by DNA sequencing of the PCR product of the target region of the genome.

### Expression in *E. coli*, purification, and refolding of recombinant proteins

The expression, purification, and refolding of recombinant proteins were carried out as described previously (40) with modifications. Briefly, after induction with isopropyl-β-D-thiogalactopyranoside (IPTG), *E. coli* BL21(DE3) cells expressing the target protein were suspended in 50 mM Tris-HCl buffer (pH 8.0) containing 3 M KCl and disrupted by sonication on ice, followed by centrifugation (13,400 × *g* for 10 min) to collect soluble and insoluble fractions. The soluble fraction containing NgVapB1H was subjected to Ni-NTA affinity chromatography on a Ni^2+^-charged Chelating Sepharose Fast Flow (GE Healthcare) column, and the elution fraction was dialyzed against 50 mM Tris-HCl buffer (pH 8.0) containing 3 M KCl overnight at 4°C, followed by centrifugation (13,400 × *g*, 10 min) to recover the soluble fraction. The insoluble fraction containing NgVapC1H was solubilized in 50 mM Tris-HCl (pH 8.0) containing 8 M urea, and then diluted 1:100 with 50 mM Tris-HCl buffer (pH 8.0) containing 3 M KCl for protein refolding. After incubation at room temperature (~25°C) for 30 min, the dilution was centrifuged at 13,400 × *g* for 15 min to recover the soluble fraction as refolded NgVapC1H. Protein concentration was determined using the Bradford method with bovine serum albumin (BSA) as a standard.

### β-Galactosidase activity assay

The β-galactosidase activity assay of strains carrying an expression plasmid for β-galactosidase was determined using *o*-nitrophenyl-*β*-D-galactopyranoside (Meilunbio, Dalian, China) as the substrate as described previously (39).

### SDS-PAGE and immunoblot analyses

Protein samples were precipitated with 20% (wt/vol) trichloroacetic acid and washed with acetone before being subjected to Tris-tricine SDS-PAGE analysis. The anti-His tag monoclonal antibody (1:10,000, Proteintech, Chicago, USA) and the horseradish peroxidase-conjugated goat anti-mouse IgG secondary antibody (1:10,000, Biodragon, Jiangsu, China) for immunoblot analysis as described previously (40), and the signals were detected with enhanced chemiluminescence (ECL) reagent (Biodragon). The images were photographed using the ChemiDoc MP imaging system (Bio-Rad, USA) with an exposure time of 0.5 or 2 min.

### Determination of mRNA level

Total RNA extraction from cells was performed using Triquick Reagent (Solarbio, China) according to the manufacturer’s instructions. Genomic DNA removal and reverse transcription were conducted using PrimeScript RT Reagent Kit with gDNA Eraser (Takara Bio Inc, Beijing) according to the manufacturer’s instructions. Using the gene-specific primers (Table S3), the qRT-PCR was carried out in a 20 μL reaction mixture as described previously (50). The qRT-PCR results were calculated using the threshold cycle (2^-ΔΔC^_T_) method (74). To quantify the transcript levels of target genes, 16S rRNA C*_T_* values were used to normalize the C*_T_* values of target transcripts.

### Electrophoretic mobility shift assay

The EMSA assay was carried out as described previously (75) with modifications. In brief, target DNA fragments for EMSA were PCR-amplified from strain J7-2 genomic DNA using the sequence-specific primers (Table S3) and purified by DNA agarose gel electrophoresis using a Gel Extraction Kit (Omega). Purified NgVapB1H (0.1–0.4 μM) was incubated with target DNA fragment (100 nM) in binding buffer (20 mM Tris-acetate [pH 6.0], 3 M KCl, 2 mM dithiothreitol, 2 mM EDTA, 15 mM MgCl_2_, 57 mM β-mercaptoethanol, 100 µg/mL BSA, and 10% [wt/vol] sorbitol) at 30°C for 30 min. Subsequently, a 20 µL reaction mixture was applied to a 6.5% native PAGE gel in 1× TBE buffer (89 mM Tris, 89 mM borate, and 2 mM EDTA, pH 8.3), and the electrophoresis was run at 120 V for 2 h at 4°C. The DNA bands on the gels were stained with ethidium bromide and visualized by the BioDocAnalyze Gel Imaging System (Analytik Jena).

### Bioinformatic analyses

The structural models of proteins were predicted by using the AlphaFold3 server (46). Molecular graphics and analyses were performed with PyMOL (Version 2.4.0, Schrödinger, LLC) with the source code available at https://github.com/schrodinger/pymol-open-source. Sequence logos were generated using WebLogo (https://weblogo.threeplusone.com/).

### Statistical analysis

All statistical analyses were performed using GraphPad Prism 9.0 (Graphpad Software, Inc., San Diego, CA, USA). Normality was assessed using the Shapiro-Wilk test. For normally distributed data, unpaired *t*-test and Welch’s *t*-test were applied for those without and with significant differences in standard deviations, respectively, to determine the significant difference between the means of the two groups; meanwhile, the F-test was used to determine the significant difference between variances of the two groups. For non-normally distributed data, the Mann-Whitney U test was used to determine the significant difference between medians of the two groups, while the Brown-Forsythe test was used to determine the significant difference between variances of two groups. In all statistical comparisons, differences were considered statistically significant at *P* < 0.05.

## Data Availability

The genome sequence of *Natrinema gari* J7-2 has been deposited in GenBank under the accession number CP003412.

## SUPPLEMENTAL MATERIAL

Supplemental material aem.00747-26-s0001.pdf Tables S1 to S3; Fig. S1 to S6.

ASM does not own the copyrights to Supplemental Material that may be linked to, or accessed through, an article. The authors have granted ASM a non-exclusive, world-wide license to publish the Supplemental Material files. Please contact the corresponding author directly for reuse.

## References

[1] Jurėnas D, Fraikin N, Goormaghtigh F, Van Melderen L. 2022. Biology and evolution of bacterial toxin-antitoxin systems. Nat Rev Microbiol 20:335–350. doi:10.1038/s41579-021-00661-1 34975154

[2] Fraikin N, Goormaghtigh F, Van Melderen L. 2020. Type II Toxin-antitoxin systems: evolution and revolutions. J Bacteriol 202:e00763-19. doi:10.1128/JB.00763-19 31932311 PMC7167474

[3] Overgaard M, Borch J, Jørgensen MG, Gerdes K. 2008. Messenger RNA interferase RelE controls relBE transcription by conditional cooperativity. Mol Microbiol 69:841–857. doi:10.1111/j.1365-2958.2008.06313.x 18532983

[4] Winther KS, Gerdes K. 2012. Regulation of enteric *vapBC* transcription: induction by VapC toxin dimer-breaking. Nucleic Acids Res 40:4347–4357. doi:10.1093/nar/gks029 22287572 PMC3378870

[5] Ruiz-Echevarría MJ, de la Cueva G, Díaz-Orejas R. 1995. Translational coupling and limited degradation of a polycistronic messenger modulate differential gene expression in the parD stability system of plasmid R1. Mol Gen Genet 248:599–609. doi:10.1007/BF02423456 7476860

[6] Nikolic N, Bergmiller T, Vandervelde A, Albanese TG, Gelens L, Moll I. 2018. Autoregulation of mazEF expression underlies growth heterogeneity in bacterial populations. Nucleic Acids Res 46:2918–2931. doi:10.1093/nar/gky079 29432616 PMC5888573

[7] Sanchez-Torres V, Hwang H-J, Wood TK. 2024. Conformational change as a mechanism for toxin activation in bacterial toxin-antitoxin systems. J Virol 98:e0151324. doi:10.1128/jvi.01513-24 39445801 PMC11575165

[8] Pandey DP, Gerdes K. 2005. Toxin-antitoxin loci are highly abundant in free-living but lost from host-associated prokaryotes. Nucleic Acids Res 33:966–976. doi:10.1093/nar/gki201 15718296 PMC549392

[9] Bendtsen KL, Brodersen DE. 2017. Higher-Order structure in bacterial VapBC Toxin-antitoxin complexes. Subcell Biochem 83:381–412. doi:10.1007/978-3-319-46503-6_14 28271484

[10] Winther K, Tree JJ, Tollervey D, Gerdes K. 2016. VapCs of *Mycobacterium tuberculosis* cleave RNAs essential for translation. Nucleic Acids Res 44:9860–9871. doi:10.1093/nar/gkw781 27599842 PMC5175351

[11] Maezato Y, Daugherty A, Dana K, Soo E, Cooper C, Tachdjian S, Kelly RM, Blum P. 2011. VapC6, a ribonucleolytic toxin regulates thermophilicity in the crenarchaeote *Sulfolobus solfataricus*. RNA 17:1381–1392. doi:10.1261/rna.2679911 21622901 PMC3138573

[12] Bhowmick A, Recalde A, Bhattacharyya C, Banerjee A, Das J, Rodriguez-Cruz UE, Albers SV, Ghosh A. 2024. Role of VapBC4 toxin-antitoxin system of *Sulfolobus acidocaldarius* in heat stress adaptation. mBio 15:e0275324. doi:10.1128/mbio.02753-24 39535218 PMC11633383

[13] Mattison K, Wilbur JS, So M, Brennan RG. 2006. Structure of FitAB from *Neisseria gonorrhoeae* bound to DNA reveals a tetramer of toxin-antitoxin heterodimers containing pin domains and ribbon-helix-helix motifs. J Biol Chem 281:37942–37951. doi:10.1074/jbc.M605198200 16982615

[14] Dienemann C, Bøggild A, Winther KS, Gerdes K, Brodersen DE. 2011. Crystal structure of the VapBC toxin-antitoxin complex from Shigella flexneri reveals a hetero-octameric DNA-binding assembly. J Mol Biol 414:713–722. doi:10.1016/j.jmb.2011.10.024 22037005 PMC3384007

[15] Kamruzzaman M, Wu AY, Iredell JR. 2021. Biological Functions of type II toxin-antitoxin systems in bacteria. Microorganisms 9:1276. doi:10.3390/microorganisms9061276 34208120 PMC8230891

[16] Cooper CR, Lewis AM, Notey JS, Mukherjee A, Willard DJ, Blum PH, Kelly RM. 2023. Interplay between transcriptional regulators and VapBC toxin-antitoxin loci during thermal stress response in extremely thermoacidophilic archaea. Environ Microbiol 25:1200–1215. doi:10.1111/1462-2920.16350 36752722 PMC10580297

[17] Cooper CR, Daugherty AJ, Tachdjian S, Blum PH, Kelly RM. 2009. Role of vapBC toxin-antitoxin loci in the thermal stress response of *Sulfolobus solfataricus*. Biochem Soc Trans 37:123–126. doi:10.1042/BST0370123 19143615 PMC2919284

[18] Mukherjee A, Wheaton GH, Counts JA, Ijeomah B, Desai J, Kelly RM. 2017. VapC toxins drive cellular dormancy under uranium stress for the extreme thermoacidophile Metallosphaera prunae. Environ Microbiol 19:2831–2842. doi:10.1111/1462-2920.13808 28585353 PMC5546240

[19] Lewis AM, Willard DJ, H. Manesh MJ, Sivabalasarma S, Albers S-V, Kelly RM. 2023. Stay or Go: Sulfolobales biofilm dispersal is dependent on a bifunctional vapb antitoxin. mBio 14:e0005323. doi:10.1128/mbio.00053-23 37036347 PMC10127717

[20] Lemmens L, Baes R, Peeters E. 2018. Heat shock response in archaea. Emerg Top Life Sci 2:581–593. doi:10.1042/ETLS20180024 33525827

[21] Arcus VL, Bäckbro K, Roos A, Daniel EL, Baker EN. 2004. Distant structural homology leads to the functional characterization of an archaeal PIN domain as an exonuclease. J Biol Chem 279:16471–16478. doi:10.1074/jbc.M313833200 14734548

[22] Bunker RD, McKenzie JL, Baker EN, Arcus VL. 2008. Crystal structure of PAE0151 from *Pyrobaculum aerophilum*, a PIN-domain (VapC) protein from a toxin-antitoxin operon. Proteins 72:510–518. doi:10.1002/prot.22048 18398909

[23] Levin I, Schwarzenbacher R, Page R, Abdubek P, Ambing E, Biorac T, Brinen LS, Campbell J, Canaves JM, Chiu H-J, et al.. 2004. Crystal structure of a PIN (PilT N-terminus) domain (AF0591) from *Archaeoglobus fulgidus* at 1.90 A resolution. Proteins 56:404–408. doi:10.1002/prot.20090 15211526

[24] Jeyakanthan J, Inagaki E, Kuroishi C, Tahirov TH. 2005. Structure of PIN-domain protein PH0500 from *Pyrococcus horikoshii*. Acta Crystallogr Sect F Struct Biol Cryst Commun 61:463–468. doi:10.1107/S1744309105012406 PMC1952296 16511069

[25] Yamaguchi Y, Nariya H, Park JH, Inouye M. 2012. Inhibition of specific gene expressions by protein-mediated mRNA interference. Nat Commun 3:607. doi:10.1038/ncomms1621 22215082

[26] Ishida Y, Inouye K, Ming O, Inouye M. 2019. A CUGGU/UUGGU-specific MazF homologue from *Methanohalobium evestigatum*. Biochem Biophys Res Commun 518:533–540. doi:10.1016/j.bbrc.2019.08.076 31445700

[27] Li M, Gong L, Cheng F, Yu H, Zhao D, Wang R, Wang T, Zhang S, Zhou J, Shmakov SA, Koonin EV, Xiang H. 2021. Toxin-antitoxin RNA pairs safeguard CRISPR-Cas systems. Science 372:eabe5601. doi:10.1126/science.abe5601 33926924

[28] Chen Z, Liu Y, Wang Y, Du X, Deng X, Xiang J, Wang Y, Wang J, Krupovic M, Du S, Chen X. 2023. A virus-borne DNA damage signaling pathway controls the lysogeny-induction switch in a group of temperate pleolipoviruses. Nucleic Acids Res 51:3270–3287. doi:10.1093/nar/gkad125 36864746 PMC10123103

[29] de Almeida JPP, Vêncio RZN, Lorenzetti APR, Caten FT-, Gomes-Filho JV, Koide T. 2019. The primary antisense transcriptome of *Halobacterium salinarum* NRC-1. Genes (Basel) 10:280. doi:10.3390/genes10040280 30959844 PMC6523106

[30] Feng J, Liu B, Zhang Z, Ren Y, Li Y, Gan F, Huang Y, Chen X, Shen P, Wang L, Tang B, Tang XF. 2012. The complete genome sequence of *Natrinema sp. J7-2*, a haloarchaeon capable of growth on synthetic media without amino acid supplements. PLoS One 7:e41621. doi:10.1371/journal.pone.0041621 22911826 PMC3402447

[31] Shen P, Chen Y. 1994. Plasmid from Halobacterium halobium and its restriction map. Yi Chuan Xue Bao 21:409–416. 7848666

[32] Tapingkae W, Tanasupawat S, Parkin KL, Benjakul S, Visessanguan W. 2010. Degradation of histamine by extremely halophilic archaea isolated from high salt-fermented fishery products. Enzyme Microb Technol 46:92–99. doi:10.1016/j.enzmictec.2009.10.011

[33] Shi W, Tang XF, Huang Y, Gan F, Tang B, Shen P. 2006. An extracellular halophilic protease SptA from a *Halophilic archaeon Natrinema* sp. J7: gene cloning, expression and characterization. Extremophiles 10:599–606. doi:10.1007/s00792-006-0003-8 16896523

[34] Xu Z, Du X, Li T, Gan F, Tang B, Tang XF. 2011. Functional insight into the C-terminal extension of Halolysin SptA from *Haloarchaeon Natrinema* sp. J7. PLoS ONE 6:e23562. doi:10.1371/journal.pone.0023562 21886797 PMC3158780

[35] Zhang Y, Wang M, Du X, Tang W, Zhang L, Li M, Wang J, Tang B, Tang XF. 2014. Chitin accelerates activation of a novel haloarchaeal serine protease that deproteinizes chitin-containing biomass. Appl Environ Microbiol 80:5698–5708. doi:10.1128/AEM.01196-14 25002433 PMC4178621

[36] Mei S, Li M, Sun Y, Deng X, Chen N, Liu Y, Yin J, Luo H, Wu Y, He D, Gan F, Tang B, Tang XF. 2022. Sec-dependent secretion of subtilase SptE in *Haloarchaea* Facilitates its proper folding and heterocatalytic processing by Halolysin SptA extracellularly . Appl Environ Microbiol 88:e0024622. doi:10.1128/aem.00246-22 35348390 PMC9040580

[37] Luo H, Qu X, Deng X, He L, Wu Y, Liu Y, He D, Yin J, Wang B, Gan F, Tang B, Tang XF. 2024. HtrAs are essential for the survival of the haloarchaeon *Natrinema gari* J7-2 in response to heat, high salinity, and toxic substances. Appl Environ Microbiol 90:e0204823. doi:10.1128/aem.02048-23 38289131 PMC10880668

[38] Du X, Li M, Tang W, Zhang Y, Zhang L, Wang J, Li T, Tang B, Tang XF. 2015. Secretion of Tat-dependent halolysin SptA capable of autocatalytic activation and its relation to haloarchaeal growth. Mol Microbiol 96:548–565. doi:10.1111/mmi.12955 25641392

[39] Li M, Yin J, Mei S, Wang X, Tang XF, Tang B. 2018. Halolysin spta, a serine protease, contributes to growth-phase transition of *Haloarchaeon Natrinema sp. J7-2*, and Its Expression Involves Cooperative Action of Multiple Cis-Regulatory Elements. Front Microbiol 9:1799. doi:10.3389/fmicb.2018.01799 30123209 PMC6085418

[40] Yin J, Liu Y, He D, Li P, Qiao M, Luo H, Qu X, Mei S, Wu Y, Sun Y, Gan F, Tang B, Tang XF. 2024. A TrmBL2-like transcription factor mediates the growth phase-dependent expression of halolysin SptA in a concentration-dependent manner in *Natrinema gari* J7-2. Appl Environ Microbiol 90:e0074124. doi:10.1128/aem.00741-24 38953660 PMC11267917

[41] Zhang H, Cui P, Lin L, Shen P, Tang B, Huang YP. 2009. Transcriptional analysis of the hsp70 gene in a haloarchaeon Natrinema sp. J7 under heat and cold stress. Extremophiles 13:669–678. doi:10.1007/s00792-009-0251-5 19448969

[42] Zhao S, Makarova KS, Zheng W, Zhan L, Wan Q, Liu Y, Gong H, Krupovic M, Lutkenhaus J, Chen X, Koonin EV, Du S. 2024. Widespread photosynthesis reaction centre barrel proteins are necessary for haloarchaeal cell division. Nat Microbiol 9:712–726. doi:10.1038/s41564-024-01615-y 38443574 PMC13379934

[43] Guan J, Chen Y, Goh YX, Wang M, Tai C, Deng Z, Song J, Ou HY. 2024. TADB 3.0: an updated database of bacterial toxin-antitoxin loci and associated mobile genetic elements. Nucleic Acids Res 52:D784–D790. doi:10.1093/nar/gkad962 37897352 PMC10767807

[44] Bertelli C, Laird MR, Williams KP, Simon Fraser University Research Computing Group, Lau BY, Hoad G, Winsor GL, Brinkman FSL. 2017. IslandViewer 4: expanded prediction of genomic islands for larger-scale datasets. Nucleic Acids Res 45:W30–W35. doi:10.1093/nar/gkx343 28472413 PMC5570257

[45] Kang SM, Kim DH, Lee KY, Park SJ, Yoon HJ, Lee SJ, Im H, Lee BJ. 2017. Functional details of the *Mycobacterium tuberculosis* VapBC26 toxin-antitoxin system based on a structural study: insights into unique binding and antibiotic peptides. Nucleic Acids Res 45:8564–8580. doi:10.1093/nar/gkx489 28575388 PMC5737657

[46] Abramson J, Adler J, Dunger J, Evans R, Green T, Pritzel A, Ronneberger O, Willmore L, Ballard AJ, Bambrick J, et al.. 2024. Accurate structure prediction of biomolecular interactions with AlphaFold 3. Nature 630:493–500. doi:10.1038/s41586-024-07487-w 38718835 PMC11168924

[47] Sullivan DM, Bobay BG, Kojetin DJ, Thompson RJ, Rance M, Strauch MA, Cavanagh J. 2008. Insights into the nature of DNA binding of AbrB-like transcription factors. Structure 16:1702–1713. doi:10.1016/j.str.2008.08.014 19000822 PMC2606041

[48] Nußbaum P, Kureisaite-Ciziene D, Bellini D, van der Does C, Kojic M, Taib N, Yeates A, Tourte M, Gribaldo S, Loose M, Löwe J, Albers S-V. 2024. Proteins containing photosynthetic reaction centre domains modulate FtsZ-based archaeal cell division. Nat Microbiol 9:698–711. doi:10.1038/s41564-024-01600-5 38443575

[49] Allers T, Barak S, Liddell S, Wardell K, Mevarech M. 2010. Improved strains and plasmid vectors for conditional overexpression of His-tagged proteins in *Haloferax volcanii*. Appl Environ Microbiol 76:1759–1769. doi:10.1128/AEM.02670-09 20097827 PMC2838008

[50] Tang W, Wu Y, Li M, Wang J, Mei S, Tang B, Tang XF. 2016. Alternative translation initiation of a haloarchaeal serine protease transcript containing two in-frame start codons. J Bacteriol 198:1892–1901. doi:10.1128/JB.00202-16 27137502 PMC4907116

[51] de Silva RT, Abdul-Halim MF, Pittrich DA, Brown HJ, Pohlschroder M, Duggin IG. 2021. Improved growth and morphological plasticity of *Haloferax volcanii*. Microbiology 167:001012. doi:10.1099/mic.0.001012 33459585 PMC8131023

[52] Wu Y, Zhang J, Wang B, Zhang Y, Li H, Liu Y, Yin J, He D, Luo H, Gan F, Tang B, Tang XF. 2023. Dissecting the Arginine and Lysine biosynthetic pathways and their relationship in *Haloarchaeon Natrinema gari J7-2* via endogenous CRISPR-Cas system-based genome editing. Microbiol Spectr 11:e0028823. doi:10.1128/spectrum.00288-23 37347159 PMC10433800

[53] Feng J, Wang J, Zhang Y, Du X, Xu Z, Wu Y, Tang W, Li M, Tang B, Tang XF. 2014. Proteomic analysis of the secretome of haloarchaeon Natrinema sp. J7-2. J Proteome Res 13:1248–1258. doi:10.1021/pr400728x 24512091

[54] Brenneis M, Hering O, Lange C, Soppa J. 2007. Experimental characterization of cis-acting elements important for translation and transcription in *Halophilic Archaea*. PLoS Genet 3:e229. doi:10.1371/journal.pgen.0030229 18159946 PMC2151090

[55] Holmes ML, Scopes RK, Moritz RL, Simpson RJ, Englert C, Pfeifer F, Dyall-Smith ML. 1997. Purification and analysis of an extremely halophilic beta-galactosidase from *Haloferax alicantei*. Biochim Biophys Acta 1337:276–286. doi:10.1016/s0167-4838(96)00174-4 9048905

[56] Christensen SK, Maenhaut-Michel G, Mine N, Gottesman S, Gerdes K, Van Melderen L. 2004. Overproduction of the Lon protease triggers inhibition of translation in *Escherichia coli*: involvement of the yefM-yoeB toxin-antitoxin system. Mol Microbiol 51:1705–1717. doi:10.1046/j.1365-2958.2003.03941.x 15009896

[57] Barros BB, Mahmoud SA, Chien P, Zeinert RD. 2020. Degradation of Lon in *Caulobacter crescentus*. J Bacteriol 203:e00344-20. doi:10.1128/JB.00344-20 33020222 PMC7723953

[58] Agarwal S, Tiwari P, Deep A, Kidwai S, Gupta S, Thakur KG, Singh R. 2018. System-wide analysis unravels the differential regulation and in vivo essentiality of virulence-associated proteins B and C toxin-antitoxin systems of *Mycobacterium tuberculosis*. J Infect Dis 217:1809–1820. doi:10.1093/infdis/jiy109 29529224

[59] Li M, Guo H, Wang L, Tao R, Song G, Cao L, Yan W, Wu Z, Liu Q, Chen Y, Gong L, Wang T, Zhang Y. 2025. A plasmid-encoded inactive toxin-antitoxin system MtvT/MtvA regulates plasmid conjugative transfer and bacterial virulence in *Pseudomonas aeruginosa*. Nucleic Acids Res 53:gkaf075. doi:10.1093/nar/gkaf075 39950345 PMC11826091

[60] Martinez-Pastor M, Tonner PD, Darnell CL, Schmid AK. 2017. Transcriptional regulation in archaea: From individual genes to global regulatory networks. Annu Rev Genet 51:143–170. doi:10.1146/annurev-genet-120116-023413 29178818

[61] Bailey SES, Hayes F. 2009. Influence of operator site geometry on transcriptional control by the YefM-YoeB toxin-antitoxin complex. J Bacteriol 191:762–772. doi:10.1128/JB.01331-08 19028895 PMC2632073

[62] Wilbur JS, Chivers PT, Mattison K, Potter L, Brennan RG, So M. 2005. *Neisseria gonorrhoeae* FitA interacts with FitB to bind DNA through its ribbon-helix-helix motif. Biochemistry 44:12515–12524. doi:10.1021/bi0511080 16156663

[63] Deter HS, Jensen RV, Mather WH, Butzin NC. 2017. Mechanisms for differential protein production in toxin-antitoxin systems. Toxins (Basel) 9:211. doi:10.3390/toxins9070211 28677629 PMC5535158

[64] Huber M, Vogel N, Borst A, Pfeiffer F, Karamycheva S, Wolf YI, Koonin EV, Soppa J. 2023. Unidirectional gene pairs in archaea and bacteria require overlaps or very short intergenic distances for translational coupling via termination-reinitiation and often encode subunits of heteromeric complexes. Front Microbiol 14:1291523. doi:10.3389/fmicb.2023.1291523 38029211 PMC10666635

[65] Brown KM, Wade JT. 2025. Translational coupling of neighboring genes in prokaryotes. J Bacteriol 207:e0025525. doi:10.1128/jb.00255-25 40919953 PMC12548453

[66] Hering O, Brenneis M, Beer J, Suess B, Soppa J. 2009. A novel mechanism for translation initiation operates in haloarchaea. Mol Microbiol 71:1451–1463. doi:10.1111/j.1365-2958.2009.06615.x 19210623

[67] Giménez MI, Cerletti M, De Castro RE. 2015. Archaeal membrane-associated proteases: insights on *Haloferax volcanii* and other haloarchaea. Front Microbiol 6:39. doi:10.3389/fmicb.2015.00039 25774151 PMC4343526

[68] Bordes P, Cirinesi AM, Ummels R, Sala A, Sakr S, Bitter W, Genevaux P. 2011. SecB-like chaperone controls a toxin-antitoxin stress-responsive system in *Mycobacterium tuberculosis*. Proc Natl Acad Sci U S A 108:8438–8443. doi:10.1073/pnas.1101189108 21536872 PMC3100995

[69] Beck IN, Arrowsmith TJ, Grobbelaar MJ, Bromley EHC, Marles-Wright J, Blower TR. 2024. Toxin release by conditional remodelling of ParDE1 from *Mycobacterium tuberculosis* leads to gyrase inhibition. Nucleic Acids Res 52:1909–1929. doi:10.1093/nar/gkad1220 38113275 PMC10899793

[70] Malakar B, Barth VC, Puffal J, Woychik NA, Husson RN. 2024. Phosphorylation of VapB antitoxins affects intermolecular interactions to regulate VapC toxin activity in *Mycobacterium tuberculosis*. J Bacteriol 206:e0023324. doi:10.1128/jb.00233-24 39315797 PMC11500542

[71] Pizzolato-Cezar LR, Spira B, Machini MT. 2023. Bacterial toxin-antitoxin systems: Novel insights on toxin activation across populations and experimental shortcomings. Curr Res Microb Sci 5:100204. doi:10.1016/j.crmicr.2023.100204 38024808 PMC10643148

[72] Ackermann M. 2015. A functional perspective on phenotypic heterogeneity in microorganisms. Nat Rev Microbiol 13:497–508. doi:10.1038/nrmicro3491 26145732

[73] Bruggeman FJ, Planqué R, Molenaar D, Teusink B. 2020. Searching for principles of microbial physiology. FEMS Microbiol Rev 44:821–844. doi:10.1093/femsre/fuaa034 33099619 PMC7685786

[74] Livak KJ, Schmittgen TD. 2001. Analysis of relative gene expression data using real-time quantitative PCR and the 2(-Delta Delta C(T)) Method. Methods 25:402–408. doi:10.1006/meth.2001.1262 11846609

[75] Johnsen U, Ortjohann M, Reinhardt A, Turner JM, Stratton C, Weber KR, Sanchez KM, Maupin-Furlow J, Davies C, Schönheit P. 2023. Discovery of a novel transcriptional regulator of sugar catabolism in archaea. Mol Microbiol 120:224–240. doi:10.1111/mmi.15114 37387308 PMC10838023

