# Supplementary material for: A haloarchaeal VapBC toxin-antitoxin system: regulatory mechanism and role in establishing population heterogeneity to facilitate survival of subpopulations under stress conditions
Source: Appl Environ Microbiol. 2026 Jul 20;92(8):e00747-26. doi: 10.1128/aem.00747-26 (PMC13488343; doi:10.1128/aem.00747-26)
Supplement: Supplementary file 1 [file aem.00747-26-s0001.pdf]

Supplementary Material

A haloarchaeal VapBC toxin-antitoxin system: regulatory mechanism and role in establishing population heterogeneity to facilitate survival of subpopulations under stress conditions

Dan He,[1] Yuqi Lin,[1] Yanyan Zhang,[1] Yang Liu,[1] Ping Li,[1] Xin Su,[1] Xiaoyi Qu,[1] Yaru Liu,[1] Xianyi Shao,[1] Hongbo Wen,[1] Hongyi Luo,[1] Yi Wu,[1] Fei Gan,[1,3] Xiao-Feng Tang,[1,3] # Bing Tang[1,2,3] #

[1]Hubei Key Laboratory of Cell Homeostasis, College of Life Sciences, Wuhan University, Wuhan, China

[2]State Key Laboratory of Virology, College of Life Sciences, Wuhan University, Wuhan, China

[3]Cooperative Innovation Center of Industrial Fermentation (Ministry of Education & Hubei Province), Wuhan, China

Running title: Characterization of a haloarchaeal TA system

#Address correspondence to Bing Tang, tangb@whu.edu.cn, or Xiao-Feng Tang, tangxf@whu.edu.cn

Keywords: haloarchaea, toxin-antitoxin system, VapBC, regulation, stress resistance

Table S1 Strains used in this study

**Strain**	**Description**	**Source**
***Escherichia coli* strains**		
DH5α	F^- φ80d/lacZΔM15 Δ(*lacZYA-argF*) U169 *deoR recA*1 *hsdR*17(rk - mk^-) *phoA supE*44 λ^- *thi*-1 *gyrA*96 *relA*1	Hanahan, 1983
JM110	*dam dcm supE*44 *hsdR*17 *thi leu rpsL1 lacY galK galT ara tonA thr tsx* (*lac-proAB*)/F' [*traD*36 *proAB*$^+$ *lacI q lacZ*ΔM15]	Yanisch-Perron et al., 1985
BL21(DE3)	F^- *omp*T $hsdS_B$ (r_B^-, m_B^-) *gal dcm* (DE3)	Novagen
***Natrinema gari* strains**		
J7-2	A subculture of *Nnm. gari* J7 (CCTCC AB 91141) lacking the plasmid pHH205	Feng et al., 2012
Δ*vapBC1*	J7-2 mutant with a deletion of *vapBC1*	This study
Δ*0581-0582*	J7-2 mutant with a deletion of *nj7g_0581-0582*	This study
Δ*2321-4368*	J7-2 mutant with a deletion of *nj7g_2321-4368*	This study
Δ*2439-2438*	J7-2 mutant with a deletion of *nj7g_2439-2438*	This study
Δ*2729-2730*	J7-2 mutant with a deletion of *nj7g_2729-2730*	This study
Δ*3796-3795*	J7-2 mutant with a deletion of *nj7g_3796-3795*	This study
Δ*3854-3855*	J7-2 mutant with a deletion of *nj7g_3854-3855*	This study
Δ*3856-3857*	J7-2 mutant with a deletion of *nj7g_3856-3857*	This study
Δ*2729*	J7-2 mutant with a deletion of *nj7g_2729*	This study
Δ*3796*	J7-2 mutant with a deletion of *nj7g_3796*	This study
Δ*3854*	J7-2 mutant with a deletion of *nj7g_3854*	This study
Δ*3856*	J7-2 mutant with a deletion of *nj7g_3856*	This study
***Hfx. volcanii* strain**		
Hfx. volcanii WFD11	Model haloarchaea without pHV2	Charlebois et al.,1987

Table S2 Plasmids used in this study

Plasmids	Description	Source
**For protein expression in *E. coli* BL21(DE3)**		
pET26b	expression vector in *E. coli*, Kan^{R}	Novagen
pET26b-*vapB1H*	pET26b-derived vector for C-terminal His-tagged NgVapB1 (NgVapB1H)	This study
pET26b-*vapB1*	pET-26b-derived vector for NgVapB1	This study
pET26b-*vapC1H*	pET26b-derived vector for C-terminal His-tagged NgVapC1 (NgVapC1H)	This study
**For construction of gene-deletion mutant**		
pYC-SHSmcs (pSHS)	*E. coli*-*Nnm. gari* J7 shuttle vector, Mev^{R}, Amp^{R}. Named as pSHS for short here.	Wang et al., 2016
pSHS-Δ*vapBC1*	pSHS-derived genome-editing vector carrying a 1-kb donor DNA containing homologous arms flanking *vapBC1* and a *vapB1*-targeting mini-CRISPR	This study
pSHS-Δ*0582-0581*	pSHS-derived genome-editing vector carrying a 1-kb donor DNA containing homologous arms flanking *nj7g_0582-0581* and a *nj7g_0582*-targeting mini-CRISPR	This study
pSHS-Δ*2321-4368*	pSHS-derived genome-editing vector carrying a 1-kb donor DNA containing homologous arms flanking *nj7g_2321-4368* and a *nj7g_2321*-targeting mini-CRISPR	This study
pSHS-Δ*2439-2438*	pSHS-derived genome-editing vector carrying a 1-kb donor DNA containing homologous arms flanking *nj7g_2439-2438* and a *nj7g_2439*-targeting mini-CRISPR	This study
pSHS-Δ*2729-2730*	pSHS-derived genome-editing vector carrying a 1-kb donor DNA containing homologous arms flanking *nj7g_2729-2730* and a *nj7g_2729*-targeting mini-CRISPR	This study
pSHS-Δ*3796-3795*	pSHS-derived genome-editing vector carrying a 1-kb donor DNA containing homologous arms flanking *nj7g_3796-3795* and a *nj7g_3796*-targeting mini-CRISPR	This study
pSHS-Δ*3854-3855*	pSHS-derived genome-editing vector carrying a 1-kb donor DNA containing homologous arms flanking *nj7g_3854-3855* and a *nj7g_3854*-targeting mini-CRISPR	This study
pSHS-Δ*3856-3857*	pSHS-derived genome-editing vector carrying a 1-kb donor DNA containing homologous arms flanking *nj7g_3856-3857* and a *nj7g_3856*-targeting mini-CRISPR	This study
pSHS-Δ*vapB1*	pSHS-derived genome-editing vector carrying a 1-kb donor DNA containing homologous arms flanking *vapB1* (without disruption of *vapC1* by retaining the -4 nt overlap) and a *vapB1*-targeting mini-CRISPR	This study

pSHS-Δ*0582*	pSHS-derived genome-editing vector carrying a 1-kb donor DNA containing homologous arms flanking *nj7g_0582* and a *nj7g_0582*-targeting mini-CRISPR	This study
pSHS-Δ*2321*	pSHS-derived genome-editing vector carrying a 1-kb donor DNA containing homologous arms flanking *nj7g_2321* and a *nj7g_2321*-targeting mini-CRISPR	This study
pSHS-Δ*2439*	pSHS-derived genome-editing vector carrying a 1-kb donor DNA containing homologous arms flanking *nj7g_2439* (without disruption of *nj7g_2438* by retaining the -4 nt overlap) and a *nj7g_2439*-targeting mini-CRISPR	This study
pSHS-Δ*2729*	pSHS-derived genome-editing vector carrying a 1-kb donor DNA containing homologous arms flanking *nj7g_2729* and a *nj7g_2729*-targeting mini-CRISPR	This study
pSHS-Δ*3796*	pSHS-derived genome-editing vector carrying a 1-kb donor DNA containing homologous arms flanking *nj7g_3796* and a *nj7g_3796*-targeting mini-CRISPR	This study
pSHS-Δ*3854*	pSHS-derived genome-editing vector carrying a 1-kb donor DNA containing homologous arms flanking *nj7g_3854* (without disruption of *nj7g_3855* by retaining the -4 nt overlap region) and a *nj7g_3854*-targeting mini-CRISPR	This study
pSHS-Δ*3856*	pSHS-derived genome-editing vector carrying a 1-kb donor DNA containing homologous arms flanking *nj7g_3856* (without disruption of *nj7g_3857* by retaining the -4 nt overlap region) and a *nj7g_3856*-targeting mini-CRISPR	This study
**For protein expression in *Hfx. volcanii* WFD11**		
pSY1	*E. coli*–*Hfx. volcanii* shuttle vector with *bop* promoter-driven *bgaH*, Amp^R, Nov^R	Yang et al., 2003
pSY1-*0346*	pSY1-derived vector carrying P_{tnaA}-*vapC1* with a C-terminal His tag coding sequence	This study
pSY1-*0581*	pSY1-derived vector carrying P_{tnaA}-*0581* with a C-terminal His tag coding sequence	This study
pSY1-*4368*	pSY1-derived vector carrying P_{tnaA}-*4368* with a C-terminal His tag coding sequence	This study
pSY1-*2438*	pSY1-derived vector carrying P_{tnaA}-*2438* with a C-terminal His tag coding sequence	This study
pSY1-*2730*	pSY1-derived vector carrying P_{tnaA}-*2730* with a C-terminal His tag coding sequence	This study
pSY1-*3795*	pSY1-derived vector carrying P_{tnaA}-*3795* with a C-terminal His tag coding sequence	This study
pSY1-*3855*	pSY1-derived vector carrying P_{tnaA}-*3855* with a C-terminal His tag coding sequence	This study
pSY1-*3857*	pSY1-derived vector carrying P_{tnaA}-*3857* with a C-terminal His tag coding sequence	This study
pSY1-*0345*	pSY1-derived vector carrying P_{tnaA}-*0345* with a C-terminal His tag coding sequence	This study
pSY1-*0582*	pSY1-derived vector carrying P_{tnaA}-*0582* with a C-terminal His tag coding sequence	This study

pSY1-*2321*	pSY1-derived vector carrying P_{tnaA}-*2321* with a C-terminal His tag coding sequence	This study
pSY1-*0345-0346*	pSY1-derived vector carrying P_{fsptA}-driven *0345* with a C-terminal His tag coding sequence and P_{tnaA}-driven *0346* with a C-terminal His tag coding sequence	This study
pSY1-*0582-0581*	pSY1-derived vector carrying P_{fsptA}-driven *0582* with a C-terminal His tag coding sequence and P_{tnaA}-driven *0581* with a C-terminal His tag coding sequence	This study
pSY1-*2321-4368*	pSY1-derived vector carrying P_{fsptA}-driven *2321* with a C-terminal His tag coding sequence and P_{tnaA}-driven *4368* with a C-terminal His tag coding sequence	This study
pSY1-*2439-2438*	pSY1-derived vector carrying P_{fsptA}-driven *2439* with a C-terminal His tag coding sequence and P_{tnaA}-driven *2438* with a C-terminal His tag coding sequence	This study
**For protein expression in *Nnm. gari* J7-2 or Δ*vapBC1***		
pSHS-P_{0566}-*vapB1*	pSHS-derived vector carrying P_{0566}-driven *vapB1* with a C-terminal His tag coding sequence	This study
pSHS-P_{tnaA}-*vapC1*	pSHS-derived vector carrying P_{tnaA}-driven *vapC1* with a C-terminal His tag coding sequence	This study
pSHS-P_{0566}-*vapB1*-P_{tnaA}-*vapC1*	pSHS-derived vector carrying P_{0566}-driven *vapB1* with a C-terminal His tag coding sequence and P_{tnaA}-driven *vapC1* with a C-terminal His tag coding sequence	This study
pSHS-P_{0566}-*vapB1*-P_{dnaK}-*vapC1*	pSHS-derived vector carrying P_{0566}-driven *vapB1* with a C-terminal His tag coding sequence and P_{dnaK}-driven *vapC1* with a C-terminal His tag coding sequence	This study
**β-Galactosidase activity assay**		
pSHS-P_{0345}-*bgaH*	pSHS-derived vector carrying P_{0345}-driven *bgaH*	This study
pSHS-$P_{0345M1r}$-*bgaH*	pSHS-derived vector carrying $P_{0345M1r}$-driven *bgaH*	This study
pSHS-$P_{0345M1f}$-*bgaH*	pSHS-derived vector carrying $P_{0345M1f}$-driven *bgaH*	This study
pSHS-$P_{0345M1fr}$-*bgaH*	pSHS-derived vector carrying $P_{0345M1fr}$-driven *bgaH*	This study
pSHS-$P_{0345M1i}$-*bgaH*	pSHS-derived vector carrying $P_{0345M1i}$-driven *bgaH*	This study
pSHS-P_{0566}-*bgaH*	pSHS-derived vector carrying P_{0566}-driven *bgaH*	This study
pSHS-P_{dnaK}-*bgaH*	pSHS-derived vector carrying P_{dnaK}-driven *bgaH*	This study
pSHS-P_{0566}-*vapB1*-P_{0345}-*bgaH*	pSHS-derived vector carrying P_{0566}-driven *vapB1* with a C-terminal His tag coding sequence and P_{0345}-driven *bgaH*	This study
pSHS-P_{0566}-*vapB1*-P_{dnaK}-*vapC1*-P_{0345} -*bgaH*	pSHS-derived vector carrying P_{0566}-driven *vapB1* with a C-terminal His tag coding sequence, P_{dnaK}-driven *vapC1* with a C-terminal His tag coding sequence, and P_{0345}-driven *bgaH*	This study

pSHS-P$_{tnaA}$-*vapB1*-P$_{tnaA}$-*vapC1*-P$_{0345}$-*bgaH*	pSHS-derived vector carrying P$_{tnaA}$-driven *vapB1* with a C-terminal His tag coding sequence, P$_{tnaA}$-driven *vapC1* with a C-terminal His tag coding sequence, and P$_{0345}$-driven *bgaH*	This study
**For start codon analysis experiment**		
pSHS-P$_{0566}$-*vapBC1*-ATG-GTG	pSHS-derived vector carrying P$_{0566}$-driven *vapBC1* operon with a C-terminal His tag coding sequence following *vapC1*. The start codons for *vapB1* and *vapC1* are ATG and GTG, respectively.	This study
pSHS-P$_{0566}$-*vapBC1*-ATG-ATG	pSHS-derived vector carrying P$_{0566}$-driven *vapBC1* operon with a C-terminal His tag coding sequence following *vapC1*. The start codons for *vapB1* and *vapC1* are ATG and ATG, respectively.	This study
pSHS-P$_{0566}$-*vapBC1*-GTG-ATG	pSHS-derived vector carrying P$_{0566}$-driven *vapBC1* operon with a C-terminal His tag coding sequence following *vapC1*. The start codons for *vapB1* and *vapC1* are GTG and ATG, respectively.	This study
pSHS-P$_{0566}$-*vapBC1*-GTG-GTG	pSHS-derived vector carrying P$_{0566}$-driven *vapBC1* operon with a C-terminal His tag coding sequence following *vapC1*. The start codons for *vapB1* and *vapC1* are GTG and GTG, respectively.	This study
pSHS-P$_{tnaA}$-*vapB1*-P$_{tnaA}$-*vapC1*-ATG-GTG	pSHS-derived vector carrying P$_{tnaA}$-driven *vapB1* and P$_{tnaA}$-driven *vapC1*, each with a C-terminal His tag coding sequence. The start codons for *vapB1* and *vapC1* are ATG and GTG, respectively.	This study
pSHS-P$_{tnaA}$-*vapB1*-P$_{tnaA}$-*vapC1*-GTG-GTG	pSHS-derived vector carrying P$_{tnaA}$-driven *vapB1* and P$_{tnaA}$-driven *vapC1*, each with a C-terminal His tag coding sequence. The start codons for *vapB1* and *vapC1* are GTG and GTG, respectively.	This study

Table S3 Primers used in this study

Primer	Sequence (5’-3’)[a]
**For construction of donors**	
vapBC1-us-F	ACCCGCCACCGACTGACGAGGGATCCGGGGAGTTTTCGGACGATCC
vapBC1-us-R	GACCGGAAACGGGTGGGTAATACTATGGTA
vapBC1-ds-F	TACCATAGTATTACCCACCCGTTTCCGGTC
vapBC1-ds-R	ATTAAATTCTTCCTAAACATTGATGGCCCGAGCAGCCGGG
0581-0582-us-F	ACCCGCCACCGACTGACGAGGGATCCGGACCGTTATCCGTCCGCCA
0581-0582-us-R	GAGATAGGGGTGATAGACGAGAAACGAACGGTCT
0581-0582-ds-F	AGACCGTTCGTTTCTCGTCTATCACCCCTATCTC
0581-0582-ds-R	CCAGCACGCTATTAAATTCTTCCTAAACATTCCCGCTCGCCGACGACG
2439-2438-us-F	ACCCGCCACCGACTGACGAGGGATCCCTCGAGCCAGATCCGCCG
2439-2438-us-R	CAATACGATGCAATACACCGAGCGACTTTCGTCAAT
2439-2438-ds-F	ATTGACGAAAGTCGCTCGGTGTATTGCATCGTATTG
2439-2438-ds-R	CCAGCACGCTATTAAATTCTTCCTAAACATCGAACCACGCGAGATACC
2729-2730-us-F	ACCCGCCACCGACTGACGAGGGATCCGGTGTCGATGAAGAGGAG
2729-2730-us-R	ACAGCAGAGGGGAGCGAGGCATGCCCGTACTTACTA
2729-2730-ds-F	TAGTAAGTACGGGCATGCCTCGCTCCCCTCTGCTGT
2729-2730-ds-R	CCAGCACGCTATTAAATTCTTCCTAAACATGACTGGTCGTCCTCTTCG
3796-3795-us-F	ACCCGCCACCGACTGACGAGGGATCCTGGGCCGGCGACGAGCGCTA
3796-3795-us-R	GTCGGTCACTACCGTCGAGTTTACCGCGCGTCCTCGAGTG
3796-3795-ds-F	ACTCGACGGTAGTGACCGACGAGACAAAT

3796-3795-ds-R	CCAGCACGCTATTAAATTCTTCCTAAACATGGCGCGCTCGATCGCGACGG
3854-3855-us-F	ACCCGCCACCGACTGACGAGGGATCCTCAGAACGTCGAACGCCT
3854-3855-us-R	GTGGAGGGACTCAAACCGAAGGCGTCGCTGAACGACT
3854-3855-ds-F	ACGAGTCGTTCAGCGACGCCTTCGGTTTGAGTCCCTCCAC
3854-3855-ds-R	CCAGCACGCTATTAAATTCTTCCTAAACATAGCTCCCGGATCTCATCCATT
3856-3857-us-F	ACCCGCCACCGACTGACGAGGGATCCCGCCAAGAAAGTACAGGAAC
3856-3857-us-R	CCGCACGGGGGAGAAAGTCATCGGCACTCTCGGTCTTCAC
3856-3857-ds-F	GTGAAGACCGAGAGTGCCGATGACTTTCTCCCCCGTGCGG
3856-3857-ds-R	CCAGCACGCTATTAAATTCTTCCTAAACATAGCCGAATTGTATCAGGGAC
2321-4368-us-F	ACCCGCCACCGACTGACGAGGGATCCTATGACCTATGATTTGATAT
2321-4368-us-R	ATGGGCGACAGCGCGGTGTATGTGTACATTGTGAGC
2321-4368-ds-F	GCTCACAATGTACACATACACCGCGCTGTCGCCCAT
2321-4368-ds-R	CCAGCACGCTATTAAATTCTTCCTAAACATCACGCTCAGCGGTCGCAA
vapB1-us-F	ACCCGCCACCGACTGACGAGGGATCCGGGGAGTTTTCGGACGATCC
vapB1-us-R	TTCGTATCGAGTATCTTCACGGTAATACTATGGTAGCACA
vapB1-ds-F	TGTGCTACCATAGTATTACCGTGAAGATACTCGATACGAA
vapB1-ds-R	CCAGCACGCTATTAAATTCTTCCTAAACATCGGAGAGTCTCATTTCGGAC
0582-us-F	ACCCGCCACCGACTGACGAGGGATCCGCGTGATGCCACTCAGGAGA
0582-us-R	GACGAGATAGGGGTGATAGAGATGGGGCCGAAACGCGTCT
0582-ds-F	AGACGCGTTTCGGCCCCATCTCTATCACCCCTATCTCGTC
0582-ds-R	CCAGCACGCTATTAAATTCTTCCTAAACATTCCCGCTCGCCGACGACG
2439-us-F	ACCCGCCACCGACTGACGAGGGATCCTCTGGCCCCGGTTTCGTCCA

2439-us-R	TGCAATACGATGCAATACACATGACCGGTGATGACCGAAC
2439-ds-F	GTTCGGTCATCACCGGTCATGTGTATTGCATCGTATTGCA
2439-ds-R	CCAGCACGCTATTAAATTCTTCCTAAACATCGAACCACGCGAGATACCCC
2729-us-F	ACCCGCCACCGACTGACGAGGGATCCGGTGTCGATGAAGAGGAG
2729-us-R	AGTCGAGAACGTACATACCGGCATGCCCGTACTTACTAAT
2729-ds-F	ATTAGTAAGTACGGGCATGCCGGTATGTACGTTCTCGACT
2729-ds-R	CCAGCACGCTATTAAATTCTTCCTAAACATCTCCCGGGCGGCCGATTCAA
3796-us-F	ACCCGCCACCGACTGACGAGGGATCCCGGGCCATCCAAGCGACCTA
3796-us-R	GTCGGTCACTACCGTCGAGTATGCCGCGTGCGGTCGTCGA
3796-ds-F	TCGACGACCGCACGCGGCATACTCGACGGTAGTGACCGAC
3796-ds-R	CCAGCACGCTATTAAATTCTTCCTAAACATGGCGCGCTCGATCGCGACGG
3854-us-F	ACCCGCCACCGACTGACGAGGGATCCTCAGAACGTCGAACGCCT
3854-us-R	AAGCTCGTATCAACGATCATGGCGTCGCTGAACGACTCGT
3854-us-F	ACGAGTCGTTCAGCGACGCCATGATCGTTGATACGAGCTT
3854-us-R	CCAGCACGCTATTAAATTCTTCCTAAACATCGATACTCGTGAGTACGTCT
3856-us-F	ACCCGCCACCGACTGACGAGGGATCCCGCCAAGAAAGTACAGGAAC
3856-us-R	TCGAGGACGAGCAGCGTCACTCGGCACTCTCGGTCTTCAC
3856-ds-F	GTGAAGACCGAGAGTGCCGA GTGACGCTGCTCGTCCTCGA
3856-ds-R	CCAGCACGCTATTAAATTCTTCCTAAACATGCGAGAGAATCCCGCTCCCG
2321-us-F	ACCCGCCACCGACTGACGAGGGATCCTATGACCTATGATTTGATAT
2321-us-R	TCAGCGATGAGCGTCGACATTATGTGTACATTGTGAGCCA
2321-ds-F	TGGCTCACAATGTACACATAATGTCGACGCTCATCGCTGA

2321-ds-R	CCAGCACGCTATTAAATTCTTCCTAAACATAGCAATGACCTGGCCCGTTG
**For construction of gene-targeting mini CRISPRs** [b]	
>Universal primers	
MC-F	ATGTTTAGGAAGAATTTAATAGCGTGCTGGAATTTCTTCGGTTGCGTTATG
MC-R	TTGCATGCCTGCAGGTCGACTCTAGAAAAAAAAAGCTTCGACCCCA
>Specific primers for the TA(A)-targeting CRISPR	
vapBC1-MC-R1	TTCTC*GCTTCGACCCCACAAGGGTCCGTCTGTAAC*CATAACGCAACCGAAAT
vapBC1-MC-R2	GTAAC**CCGGAGGAACTCGACCGGGAACTCGAGCGGTTTCTC**GCTTC
vapBC1-MC-R3	AAAAAAAA*GCTTCGACCCCACAAGGGTCCGTCTGTAAC*CCGGAGGAACTC
0582-0581-MC-R1	ACCGAGTTCC*GCTTCGACCCCACAAGGGTCCGTCTGTAAC*CATAACGCAACCGAA
0582-0581-MC-R2	GTAAC**GTGACGAACTCAATCTCGAGGACGGGACCGAGTTCC**GCTTC
0582-0581-MC-R3	AAAAAAAA*GCTTCGACCCCACAAGGGTCCGTCTGTAAC*GTGACGAACTCA
2439-2438-MC-R1	CATCGCCCTC*GCTTCGACCCCACAAGGGTCCGTCTGTAAC*CATAACGCAACCGAA
2439-2438-MC-R2	GTAAC**CGCGGTGCGCGCGAGGAACTGATCCGCATCGCCCTC**GCTTC
2439-2438-MC-R3	AAAAAAAA*GCTTCGACCCCACAAGGGTCCGTCTGTAAC*CGCGGTGCGCGC
2729-2730-MC-R1	GCTCGATGAC*GCTTCGACCCCACAAGGGTCCGTCTGTAAC*CATAACGCAACCGAA
2729-2730-MC-R2	GTAAC**GGCGCGCCGGGACAGTTCCTCGTCGGGCTCGATGAC**GCTTC
2729-2730-MC-R3	AAAAAAAA*GCTTCGACCCCACAAGGGTCCGTCTGTAAC*GGCGCGCCGGGA
3796-3795-MC-R1	GGTCCGAGTG*GCTTCGACCCCACAAGGGTCCGTCTGTAAC*CATAACGCAACCGAA
3796-3795-MC-R2	GTAAC**CGGTGGAACGTCGAAGACGACGGAGCGGTCCGAGTG**GCTTC
3796-3795-MC-R3	AAAAAAAA*GCTTCGACCCCACAAGGGTCCGTCTGTAAC*CGGTGGAACGTC
3854-3855-MC-R1	ACACATCGCG*GCTTCGACCCCACAAGGGTCCGTCTGTAAC*CATAACGCAACCGAA

3854-3855-MC-R2	GTAAC**CATCTCGCTCACCTGGTCGTCGTCGAACACATCGCG**GCTTC
3854-3855-MC-R3	AAAAAAAA*GCTTCGACCCCACAAGGGTCCGTCTGTAAC*CATCTCGCTCAC
3856-3857-MC-R1	CTCTTATCGT*GCTTCGACCCCACAAGGGTCCGTCTGTAAC*CATAACGCAACCGAA
3856-3857-MC-R2	GTAAC**CGTCTGAATGTGCCAACAAGCCGTTACTCTTATCGT**GCTTC
3856-3857-MC-R3	AAAAAAAA*GCTTCGACCCCACAAGGGTCCGTCTGTAAC*CGTCTGAATGTG
2321-4368-MC-R1	TTTCCTGCGT*GCTTCGACCCCACAAGGGTCCGTCTGTAAC*CATAACGCAACCGAA
2321-4368-MC-R2	GTAAC**CTCGTCGTCGTAGTAGGCAGCCGCGATTTCCTGCGT**GCTTC
2321-4368-MC-R3	AAAAAAAA*GCTTCGACCCCACAAGGGTCCGTCTGTAAC*CTCGTCGTCGTA
**For PCR screening and DNA sequencing**	
vapBC1-out-F	CATCATCTCGGGGGAGTTTT
vapBC1-out-R	ATGTGATGGCCCGAGCAGCC
0582-0581-out-F	TGGCCGGCACGGACCGTTAT
0582-0581-out-R	TCCCGCTCGCCGACGACGCGGT
2439-2438-out-F	CTCGAGCCAGATCCGCCGCCGGCTCAAGCTGCTGAAA
2439-2438-out-R	CGAACCACGCGAGATACCCCATCGCAAA
2729-2730-out-F	CAGCGTGGTAAAGGAGTGAT
2729-2730-out-R	TCGTCCTCTTCGGGGCGCTTTTCGTGATTT
3796-3795-out-F	TGGGCCGGCGACGAGCGCTA
3796-3795-out-R	GGCGCGCTCGATCGCGACGG
3854-3855-out-F	TTACCCACGACTCGAGAGTT
3854-3855-out-R	GCTCCCGGATCTCATCCATT
3856-3857-out-F	CGCCAAGAAAGTACAGGAAC

3856-3857-out-R	CCGCACGGGGGAGAAAGTCA
2321-4368-out-F	TATGACCTATGATTTGATATCATTAATT
2321-4368-out-R	CACGCTCAGCGGTCGCAATCGTCTCA
vapBC1-in-F	CATCATCTCGGGGGAGTTTT
vapBC1-in-R	ACGCTGACGTTGGCGTACTT
0582-0581-in-F	AGTATCGCTTGTCCCACTCATT
0582-0581-in-R	TCCCGCTCGCCGACGACGCGGT
2439-2438-in-F	CGTGCAGTGACGTTTTCCAT
2439-2438-in-R	CGAACCACGCGAGATACCCCATCGCAAA
2729-2730-in-F	CAGCGTGGTAAAGGAGTGAT
2729-2730-in-R	GCGCTGGACGACAATGTAGT
3796-3795-in-F	TCGACCCGATCGGTACTCAT
3796-3795-in-R	GGCGCGCTCGATCGCGACGG
3854-3855-in-F	TTACCCACGACTCGAGAGTT
3854-3855-in-R	CCACCGATGAGTCGGTCCAC
3856-3857-in-F	CGCCAAGAAAGTACAGGAAC
3856-3857-in-R	ACTGTCGCGTTCGGATTCTA
2321-4368-in-F	TATGACCTATGATTTGATATCATTAATT
2321-4368-in-R	CGGAACTGAACGCGCTTTTT
**For monitoring the plasmid curing**	
s1-f	TACCCGAATCAAATGGGAGT
s1-r	GCATTGTGCGGATCTTGCTT

**For construction of expression plasmids to produce toxins in *Hfx. volcanii* WFD11[c]**	
**>Universal primers**	
pSY1-P_{tnaA}-F	CTCACTATAGGGCGAATTGGGTACCGTTCTCGTCGCGCTCTCG
pSY1-P_{tnaA}-R	ATGCGCAATAGGTCCGCGAAT
t.syn-R	CGCGCCGAAAAATGCGATGGTCCAGAGGTGCGGCCCTAGTGATGGTGATGGTGATG
pSY1-T-R	AGCTAAGCTGCAGTGATGCCCATGGCGCGCCGAAAAATGCGAT
**> Specific primers for toxin expression**	
pSY1-0346-F	TTACTTCACATTCGCGGACCTATTGCGCATGTGAAGATACTCGATACG
pSY1-0346-R	CTAGTGATGGTGATGGTGATGTTCGACGTGCTCGAGTGT
pSY1-0581-F	TTACTTCACATTCGCGGACCTATTGCGCATATGGGGCCGAAACGCGTC
pSY1-0581-R	CTAGTGATGGTGATGGTGATGCGATTCGTTGACGGTGTG
pSY1-2438-F	TTACTTCACATTCGCGGACCTATTGCGCATATGACCGGTGATGACCGA
pSY1-2438-R	CTAGTGATGGTGATGGTGATGCCGGTCGGAGGCGGCATC
pSY1-2730-F	TTACTTCACATTCGCGGACCTATTGCGCATATGTACGTTCTCGACTCC
pSY1-2730-R	CTAGTGATGGTGATGGTGATGCGACGGGTTCTTCCGGGC
pSY1-3795-F	TTACTTCACATTCGCGGACCTATTGCGCATTTGGCGACAGCGAAATCG
pSY1-3795-R	CTAGTGATGGTGATGGTGATGGTGCGGATCGGACGGGTT
pSY1-3855-F	TTACTTCACATTCGCGGACCTATTGCGCATGTGGACGTTATCGATGAT
pSY1-3855-R	CTAGTGATGGTGATGGTGATGCCGGTAGCCTTCGTGGGT
pSY1-3857-F	TTACTTCACATTCGCGGACCTATTGCGCATGTGACGCTGCTCGTCCTC
pSY1-3857-R	CTAGTGATGGTGATGGTGATGGTAGGGATCGTACGTAGC
pSY1-4368-F	TTACTTCACATTCGCGGACCTATTGCGCATATGTCGACGCTCATCGCT

pSY1-4368-R	CTAGTGATGGTGATGGTGATGGGGTTCCTCAAGCAATGA
**For construction of expression plasmids to produce antitoxins in *Hfx. volcanii* WFD11[d]**	
**>Universal primers**	
pSY1-P_{fsptA}-F	CTCACTATAGGGCGAATTGGGTACCTTTAGGAAGAATTTAATAGCATGCT
t.l11e-F	CACCACCACCACCACCACTGAGACTTCGACGACTACTTCGA
pSY1-A-R	AGCTAAGCTGCAGTGATGCCCATGGGGGTCGAATCGGGTCGGTGG
**> Specific primers for antitoxin expression**	
pSY1-0345-F	GAAGAATTTAATAGCATGCTGGAATTATTTCGGTTGCGTTATGAAGTACGCCAACGTC
pSY1-0345-R	TCAGTGGTGGTGGTGGTGGTGCGTCTGTTCCGCGAATTCGTC
pSY1-0582-F	GAAGAATTTAATAGCATGCTGGAATTATTTCGGTTGCGTTATGAGTGGGACAAGCGAT
pSY1-0582-R	TCAGTGGTGGTGGTGGTGGTGCCGACCACCGAACGTCGCGT
pSY1-2439-F	GAAGAATTTAATAGCATGCTGGAATTATTTCGGTTGCGTTATGGAAAACGTCACTGCA
pSY1-2439-R	TCAGTGGTGGTGGTGGTGGTGTCGGAGCGCGTCGGCATCGG
pSY1-2321-F	GAAGAATTTAATAGCATGCTGGAATTATTTCGGTTGCGTTATGAGTAGCGACAAAAAG
pSY1-2321-R	TCAGTGGTGGTGGTGGTGGTGTGCGTCGGCGACCTCGTCGA
**For the construction of expression plasmids to produce toxin and antitoxin proteins in *Hfx. volcanii* WFD11.**	
pSY1-P_{fsptA}-F	CTCACTATAGGGCGAATTGGGTACCTTTAGGAAGAATTTAATAGCATGCT
pSY1-T-R	AGCTAAGCTGCAGTGATGCCCATGGCGCGCCGAAAAATGCGAT
pSY1-AT-F	CCACCGACCCGATTCGACCCGTTCTCGTCGCGCTCTCGAA
pSY1-AT-R	TTCGAGAGCGCGACGAGAACGGGTCGAATCGGGTCGGTGG
**Primers required for electrophoretic mobility shift assay**	
P_{0345}-F	CACGACTAGGGCGTGACAGA

P_{0345}-R	GGTAATACTATGGTAGCACAAAGTCATAAAG
$P_{0345M1r}$-R	GTGCCGCATATGGTAGCACAAAGTCATAAAGATA
$P_{0345M1f}$-R	GGTAATACTATGTGCTACAAAAGTCATAAAGATA
$P_{0345M1fr}$-R	GTGCCGCATATGTGCTACAAAAGTCATAAAGATA
$P_{0345M1i}$-R	GGTAATACGCGTGTAGCACAAAGTCATAAAGA
**For construction of expression plasmids to assay β-galactosidase activity**[e]	
>**Universal primers**	
bgaH-F	ATGACAGTTGGTGTCTGCTATT
bgaH-Xba1-R	TTGCATGCCTGCAGGTCGACTCTAGATCACTCGGACGCGAGTCC
>**Specific primers for the promoter P_{0345} and its mutants**	
P_{0345}-BamH1-F	CCCGCCACCGACTGACGAGGGATCCCACGACTAGGGCGTGACA
P_{0345}-*bgaH*-R	CTCCGGGAAATAGCAGACACCAACTGTCATGGTAATACTATGGTAGCA
$P_{0345M1fr}$-*bgaH*-R	CTCCGGGAAATAGCAGACACCAACTGTCATGTGCCGCATATGGTAGCACAAA
$P_{0345M1f}$-*bgaH*-R	CTCCGGGAAATAGCAGACACCAACTGTCATGGTAATACTATGTGCTACAAAA
$P_{0345M1fr}$-*bgaH*-R	CTCCGGGAAATAGCAGACACCAACTGTCATGTGCCGCATATGTGCTACAA
$P_{0345M1i}$-*bgaH*-R	GGAAATAGCAGACACCAACTGTCATGGTAATACGCGTGTAGCACAAAGTCATAAAGA
> **Specific primers for plasmid pSHS-P_{0566}-*vapB1*-P_{dnaK}-*vapC1*-P_{0345}-*bgaH***	
P_{0566}-BamH1-F	CCCGCCACCGACTGACGAGGGATCCTGCCGATCGTCGTCGGCTC
P_{0566}-*bgaH*-R	TAGCAGACACCAACTGTCATCACCGGGGTTTGGGCCTGAC
P_{dnaK}-BamH1-F	CCCGCCACCGACTGACGAGGGATCCGGTCGGCCGGGTGCCGGG
P_{dnaK}-*bgaH*-R	TAGCAGACACCAACTGTCATCTTGCCCGCAAATACCGTCT
P_{0566}-*vapB1*- P_{dnaK}-*vapC1*-*bgaH*-R1	CTGACGTTGGCGTACTTCATCACCGGGGTTTGGGCCTGAC
P_{0566}-*vapB1*- P_{dnaK}-*vapC1*-*bgaH*-F1	GTCAGGCCCAAACCCCGGTGATGAAGTACGCCAACGTCAG

P_{0566}-*vapB1*- P_{dnaK}-*vapC1*-*bgaH*-R2	AAAAAAAATTAGTGGTGGTGGTGGTGGTGCGTCTGTTCCGCGAATTCGTC
P_{0566}-*vapB1*- P_{dnaK}-*vapC1*-*bgaH*-F2	GCACCACCACCACCACCACTAATTTTTTTTGGTCGGCCGGGTGCCGGGTC
P_{0566}-*vapB1*- P_{hsp70}-*vapC1*-*bgaH*-R3	TTCGTATCGAGTATCTTCACCTTGCCCGCAAATACCGTCT
P_{0566}-*vapB1*- P_{dnaK}-*vapC1*-*bgaH*-F3	GTGAAGATACTCGATACGAA
P_{0566}-*vapB1*- P_{dnaK}-*vapC1*-*bgaH*-R4	AAAAAAAATTAGTGATGGTGATGGTGATGTTCGACGTGCTCGAGTGTGAT
P_{0566}-*vapB1*- P_{dnaK}-*vapC1*-*bgaH*-F4	CATCACCATCACCATCACTAATTTTTTTTCACGACTAGGGCGTGACAGAG
P_{0566}-*vapB1*- *bgaH*-R1	CTCTGTCACGCCCTAGTCGTGAAAAAAAATTAGTGGTGGTGGTGGTGGTG
P_{0566}-*vapB1*- *bgaH*-F1	CACCACCACCACCACCACTAATTTTTTTTCACGACTAGGGCGTGACAGAG
**> Specific primers for plasmid pSHS-P_{tnaA}-*vapB1*-P_{tnaA}-*vapC1*-P_{0345}-*bgaH***	
P_{tnaA}-BamH1-F	CCCGCCACCGACTGACGAGGGATCCGTTCTCGTCGCGCTCTCGA
P_{tnaA}-*vapB1*-P_{tnaA}-*vapC1*-*bgaH*-F1	TTCGCGGACCTATTGCGCATATGAAGTACGCCAACG
P_{tnaA}-*vapB1*-P_{tnaA}-*vapC1*-*bgaH*-R1	CGTTGGCGTACTTCATATGCGCAATAGGTCCGCGAA
P_{tnaA}-*vapB1*-P_{tnaA}-*vapC1*-*bgaH*-F2	CACCACCACCACCACCACTAATTTTTTTTGTTCTCGTCGCGCTCTCGAAG
P_{tnaA}-*vapB1*-P_{tnaA}-*vapC1*-*bgaH*-R2	AAAAAAAATTAGTGGTGGTGGTGGTGGTGCGTCTGTTCCGCGAATTCGTC
P_{tnaA}-*vapB1*-P_{tnaA}-*vapC1*-*bgaH*-F3	GTGAAGATACTCGATACGAA
P_{tnaA}-*vapB1*-P_{tnaA}-*vapC1*-*bgaH*-R3	TTCGTATCGAGTATCTTCACATGCGCAATAGGTCCGCGAA
**Primers required for the start codon analysis experiment**	
P_{0566}-BamH1-F	CCCGCCACCGACTGACGAGGGATCCTGCCGATCGTCGTCGGCTC
P_{0566}-R	CACCGGGGTTTGGGCCTGAC
vapB1-R	GTCTGTTCCGCGAATTCGTC
vapC1-Xba1-R	GCCTGCAGGTCGACTCTAGAAAAAAAAATTAGTGATGGTGATGGTGATG
vapB1-ATG-F	GTCAGGCCCAAACCCCGGTGATGAAGTACGCCAACGTCAG

vapB1-GTG-F	GTCAGGCCCAAACCCCGGTGGTGAAGTACGCCAACGTCAG
vapC1-ATG-F	GACGAATTCGCGGAACAGACATGAAGATACTCGATACGAA
vapC1-GTG-F	GACGAATTCGCGGAACAGACGTGAAGATACTCGATACGAA
P_{tnaA}-BamH1-F	CCCGCCACCGACTGACGAGGGATCCTGCCGATCGTCGTCGGCTC
P_{tnaA}-*vapB1*-GTG-F1	GTGAAGTACGCCAACGTCAG
P_{tnaA}-*vapB1*-GTG-R1	CTGACGTTGGCGTACTTCACATGCGCAATAGGTCCGCGAA
**For qRT-PCR**	
pSHSQRT-f	GCGGAAATACCCGAACACCAAG
pSHSQRT-r	CTCGCCACAGCAGTCGCAGAT
*vapB1*QRT-f	CGGCGTCTACACGAACAAAA
*vapB1*QRT-r	TTCGAAAGCTGCTCGTCACT
*vapC1*QRT-f	ATGGTTCAGGAAGCGCTCA
*vapC1*QRT-r	TTCGGTTGAATGGTCAGCAC
**For construction of expression plasmids in strain J7-2 and Δ*vapBC1***	
P_{tnaA}-BamH1-F	CCCGCCACCGACTGACGAGGGATCCGTTCTCGTCGCGCTCTCGA
P_{0566}-BamH1-F	CCCGCCACCGACTGACGAGGGATCCTGCCGATCGTCGTCGGCTC
vapB1-Xba1-R	GCCTGCAGGTCGACTCTAGAAAAAAAAATTAGTGGTGGTGGTGGTGGTG
vapC1-Xba1-R	GCCTGCAGGTCGACTCTAGAAAAAAAAATTAGTGATGGTGATGGTGATG
P_{sptA}-BamH1-F	CCCGCCACCGACTGACGAGGGATCCATGTTTAGGAAGAATTTAA
P_{sptA}-R	AGGGTCGTCAACGTTCGTATCGTTACTCATAACGCAACCGAAGAAATTCC
lonB-F	ATGAGTAACGATACGAAC
lonB-R	AAAAAAAATTAGTGATGGTGATGGTGATGCTGCGGGCTCGGGTTCG

**For construction of expression plasmid for recombinant proteins in *E. coli***	
vapB1-F	AACTTTAAGAAGGAGATATACATATGAAGTACGCCAACGTCAGCGT
vapB1-R	ATCCGAATTAATTCCGATATCCATGGTCACGTCTGTTCCGCGAATT
vapB1-CH-R	AATTCCGATATCCATGGTCAGTGATGGTGATGGTGATGCGTCTGTTCCGCGAATTCGT
vapC1-F	AACTTTAAGAAGGAGATATACATATGAAGATACTCGATACGAATCT
vapC1-CH-R	ATTAATTCCGATATCCATGGCTAGTGATGGTGATGGTGATGTTCGACGTGCTCGAGTGT

[a]Restriction sites are underlined. The mutated nucleotides are shown in red. The His6 tag-coding DNA sequences are shown in green. The transcription terminators are shown in orange. The spacers are in boldface. The italicized sections indicate the repeats.

[b]The universal primers were used repeatedly for constructing different mini-CRISPRs, and the specific primers were used to construct corresponding mini CRISPRs.

[c]Universal primers were used repeatedly for constructing the P_{tnaA} promoter, the t.syn terminator, and for vector ligation, while specific primers were used for constructing the corresponding open reading frames.

[d]Universal primers were used repeatedly for constructing the P_{fsptA} promoter, the t.l11e terminator, and for vector ligation, while specific primers were used for constructing the corresponding open reading frames.

[e]Universal primers were used repeatedly for *bgaH* construction, while specific primers were used for constructing the corresponding promoter and *vapBC1* ORFs.

References

Charlebois RL, Lam WL, Cline SW, Doolittle WF. 1987. Characterization of pHV2 from *Halobacterium volcanii* and its use in demonstrating transformation of an archaebacterium. Proc Natl Acad Sci U S A 84:8530-8534.

Feng J, Liu B, Zhang Z, Ren Y, Li Y, Gan F, Huang Y, Chen X, Shen P, Wang L, Tang B,Tang XF. 2012. The complete genome sequence of *Natrinema* sp. J7-2, a haloarchaeon capable of growth on synthetic media without amino acid supplements. PLoS One 7:e41621.

Hanahan D. 1983. Studies on transformation of *Escherichia coli* with plasmids. J Mol Biol 166: 557-580.

Wang Y, Sima L, Lv J, Huang S, Liu Y, Wang J, Krupovic M,Chen X. 2016. Identification, characterization, and application of the replicon region of the halophilic temperate Sphaerolipovirus SNJ1. J Bacteriol 198:1952-1964.

Yang Y, Huang Y P,Shen P. 2003. The 492-bp RM07 DNA fragment from the halophilic archaea confers promoter activity in all three domains of life. Curr Microbiol 47:388-394.

Yanisch-Perron C, Vieira J, Messing J. 1985. Improved M13 phage cloning vectors and host strains: nucleotide sequences of the M13mp18 and pUC19 vectors. Gene 33:103-119

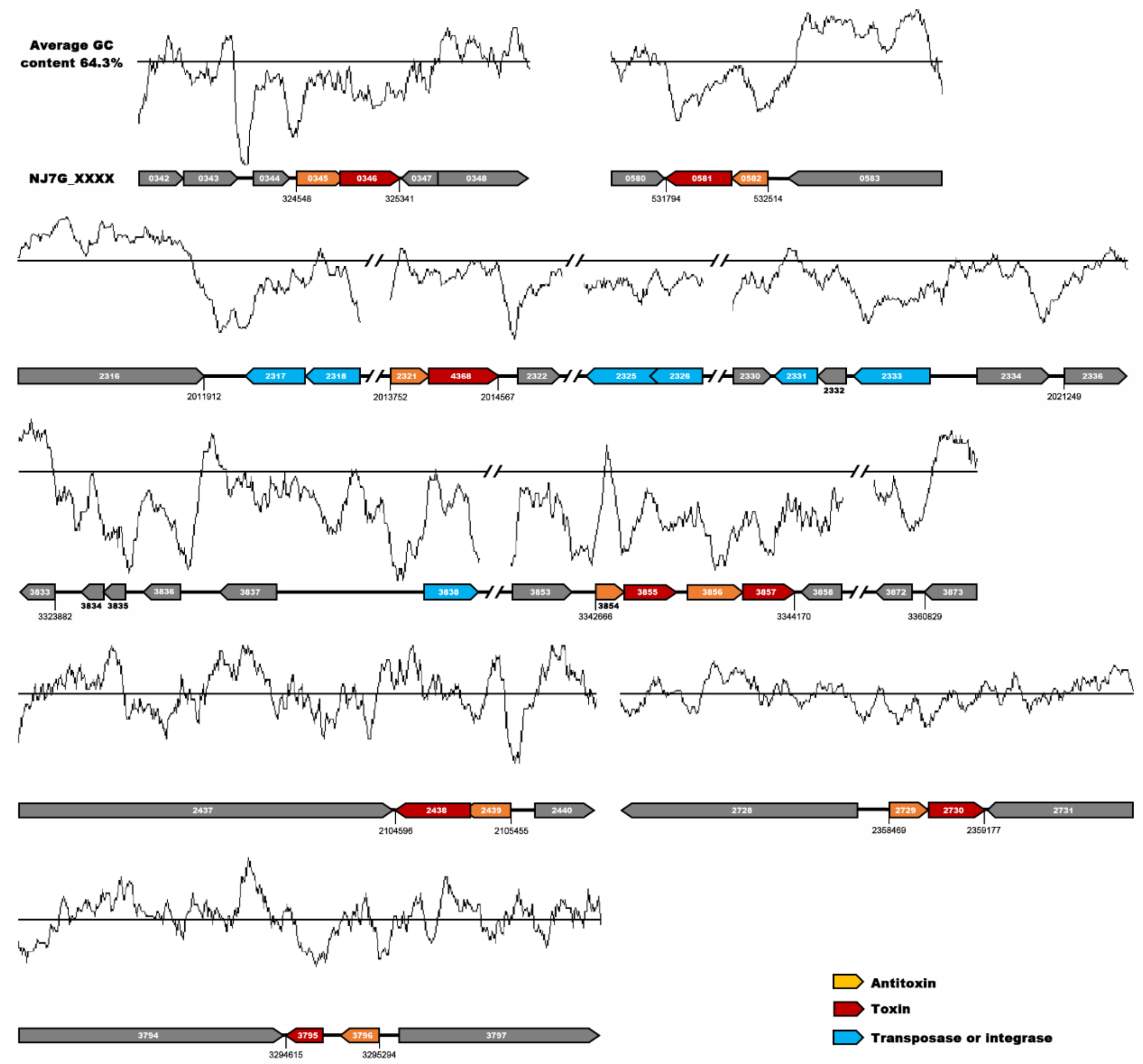

FIG S1 Overview of putative VapBCs in *Natrinema gari* J7-2. The genes are drawn to scale as arrows, and their accession numbers and GC contents are shown.

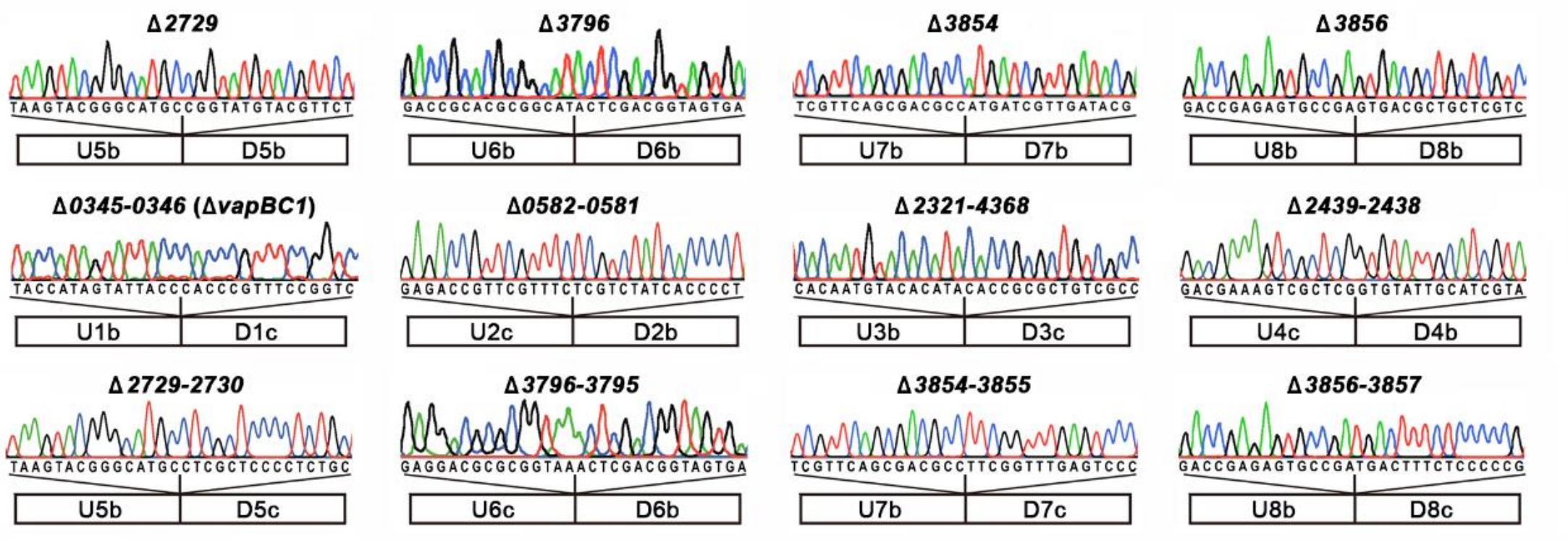

FIG S2 Representatives of chromatographs of DNA sequencing results of target mutant strains.

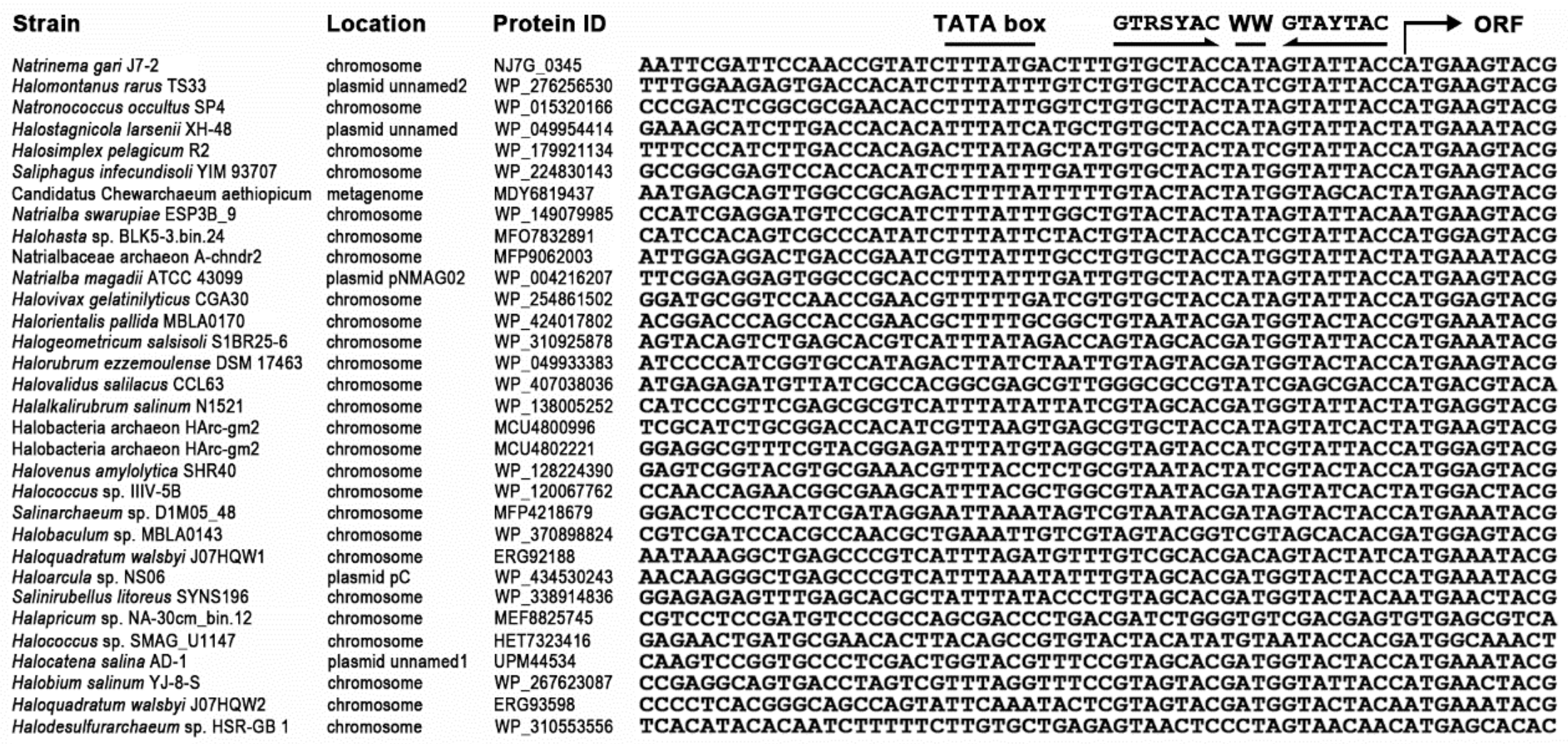

FIG S3 Sequence alignment of promoter regions of NgVapBC1 and its 31 homologs form haloarchaea. The conserved inverted repeat motifs of PPS1 are indicated by arrows. The TATA box and WW motif are underlined.

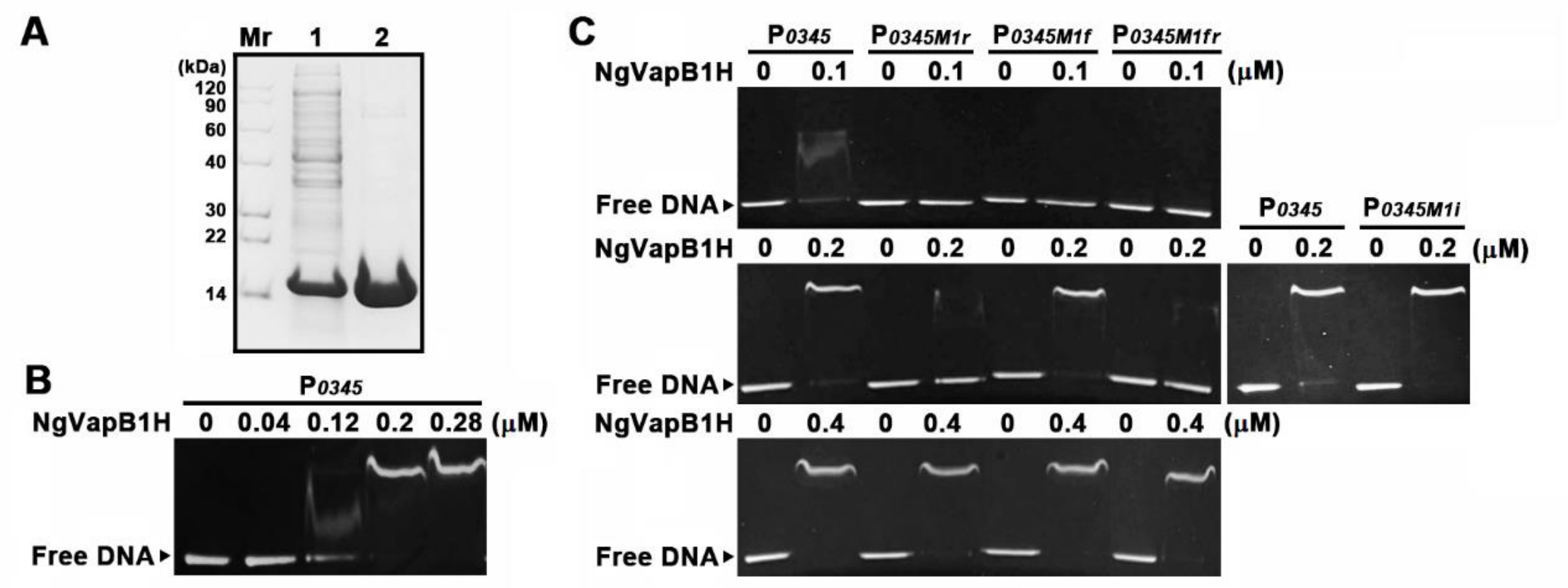

FIG S4 Binding of NgVapB1 to the promoter P_{0345} and its mutants. (A) SDS-PAGE analysis of crude (lane 1) and purified (lane 2) samples of NgVapB1H. (B and C) EMSA assay. The DNA fragment (100 nM) of target promoter region was incubated 30°C for 30 min with different concentrations of NgVapB1H in the presence of 3 M KCl, followed by native PAGE and visualization with ethidium bromide staining.

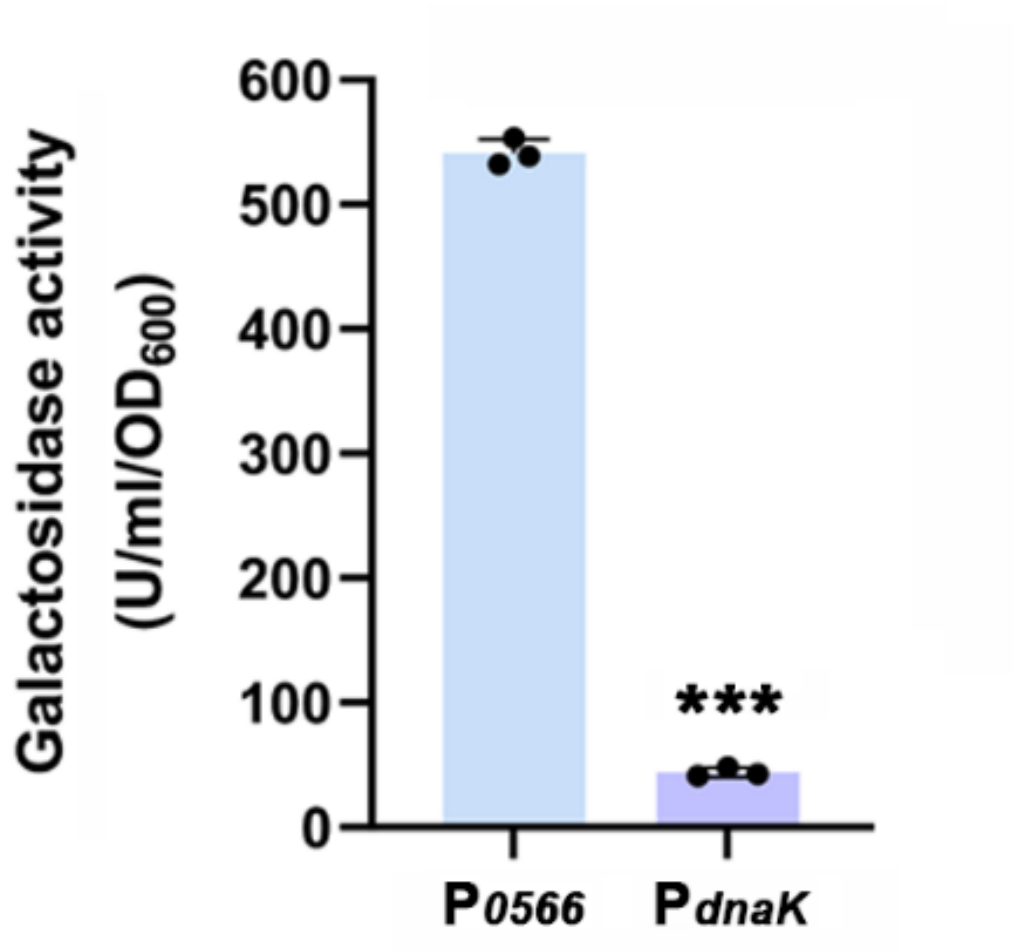

FIG S5 Comparison of promoter activities between P_{0566} and P_{dnaK}. Strain J7-2 carrying the blank vector (pSHS) or the expression plasmid for BgaH, driven by P_{0566} or P_{dnaK}, was grown at 37°C to mid-exponential phase in 23% MGM, followed by BgaH activity assay. The data are expressed as means ± SDs from biological triplicates (***, P < 0.001).

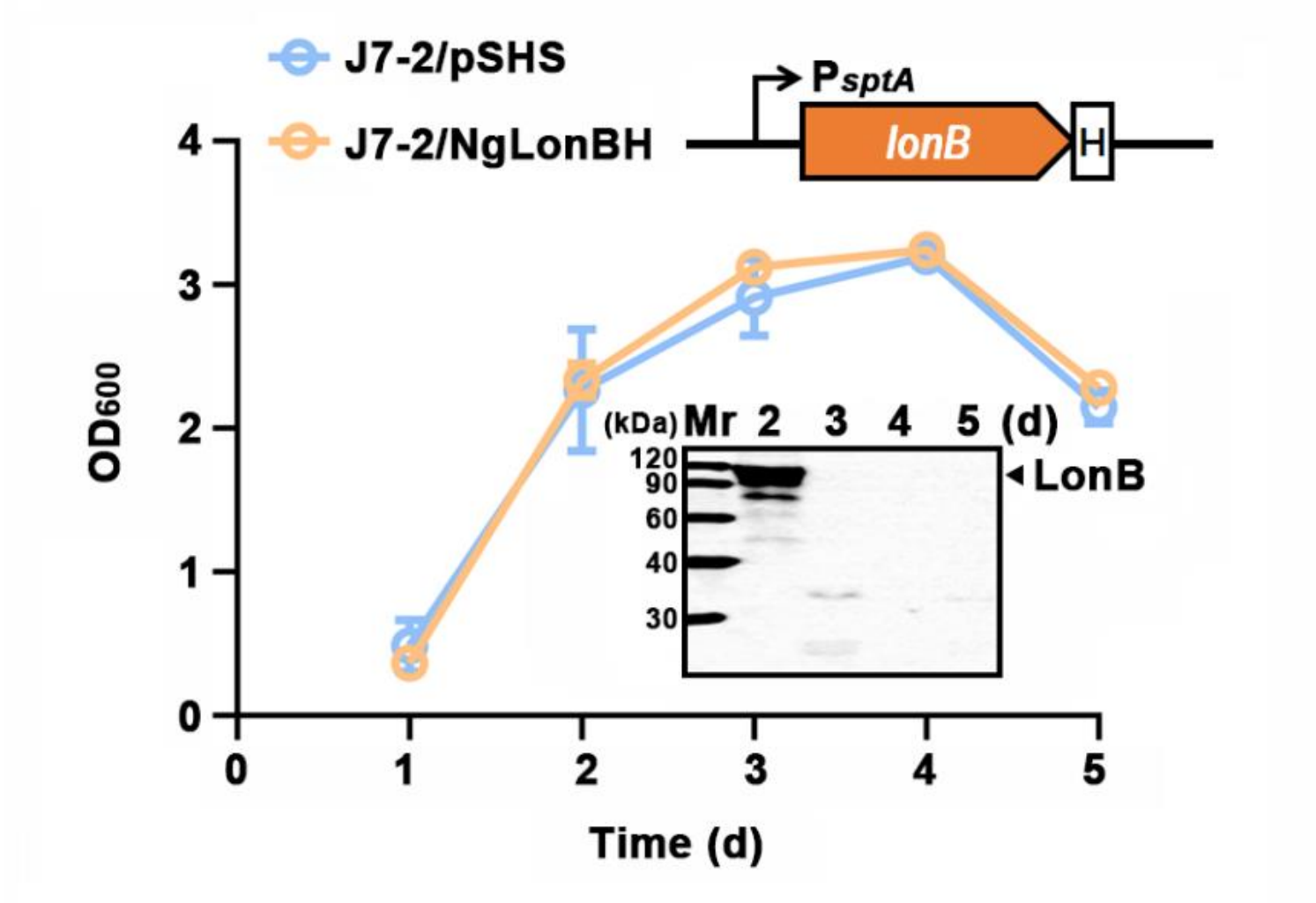

FIG S6 Overexpression of LonB protease in strain J7-2. The target strains were grown in 23% MGM with trace elements (TE) (de Silva RT et al.,, 2021) at 37°C. At the indicated time points, samples were taken and subjected to OD600 measurement and anti-His-tag immunoblot analysis (inset). Schematic representation of promoter (P_{sptA})-preceded *lonB* of strain J7-2 on the expression plasmid is shown, and the data are expressed as means ± SDs from biological triplicates. The arrowhead indicates the position of NgLonBH.

Reference

de Silva RT, Abdul-Halim MF, Pittrich DA, Brown HJ, Pohlschroder M, Duggin IG. 2021. Improved growth and morphological plasticity of *Haloferax volcanii*. Microbiology (Reading) 167:001012.
